# Purchase of food from the new staple food basket in low-income Brazilian households

**DOI:** 10.11606/s1518-8787.2025059006780

**Published:** 2025-12-08

**Authors:** Marcos Anderson Lucas da Silva, Lucas Braga Rodrigues, Samuel Almeida Brito, Luisa Gazola Lage, Maria Laura da Costa Louzada

**Affiliations:** I Universidade de São Paulo. Faculdade de Saúde Pública. Programa de Pós-Graduação em Nutrição em Saúde Pública. São Paulo, SP, Brasil; II Universidade de São Paulo. Núcleo de Pesquisas Epidemiológicas em Nutrição e Saúde. São Paulo, SP, Brasil; III Universidade Federal do Ceará. Instituto de Cultura e Artes. Programa de Pós-Graduação em Gastronomia. Fortaleza, CE, Brasil; IV Universidade de Lisboa. Instituto de Ciências Sociais. Programa de Pós-Graduação em Ciências da Sustentabilidade. Lisboa, Portugal

**Keywords:** Staple Food Basket, Healthy Eating, Food Purchase, Bolsa Família, Low Income

## Abstract

**OBJECTIVE:**

To describe the purchase of foods included in the new staple food basket by low-income households in Brazil, from 2017 to 2018.

**METHODS:**

Data on household food purchases were obtained from the 2017–2018 Household Budget Survey, conducted by the *Instituto Brasileiro de Geografia e Estatística* (Brazilian Institute of Geography and Statistics). Data from 13,706 low-income households (< ½ minimum wage *per capita*) were analyzed. Foods were identified based on an ordinance issued by the Ministry of Social Development and Fight Against Hunger, which specifies the composition of the new staple food basket. Estimates were expressed as a percentage of caloric intake.

**RESULTS:**

Foods introduced into the new staple food basket accounted for 84.1% of the total calories purchased by low-income households in Brazil, from 2017 to 2018, with significant participation in rural areas (88.0%) and in the North (88.2%) and Northeast (85.9%) regions. Among food groups, cereals (33.5%), sugars and oils/fats (21.3%), and meats and eggs (11.6%) presented the highest contributions. Rice, corn, and other grains contributed 19.7%, whereas beans accounted for 4.7% of calories. Poultry meat and beef were the most purchased foods in the meat and egg group, representing 5.0% and 4.0% of calories, respectively. Fruits contributed 1.9% and vegetables 0.7% of calories. We observed no major differences in food purchase regarding the race/skin color of the head of the household.

**CONCLUSION:**

Analysis shows that in the 2017–2018 period, the foods included in the new staple food basket became the basis of low-income households’ diet, reinforcing the cultural viability of its implementation.

## INTRODUCTION

The national staple food basket is a set of essential foods that aim to meet basic nutritional needs. Established in 1938 during Getúlio Vargas’ government, the basket was conceived as a basis for calculating the minimum wage and specified the types and quantities necessary for the subsistence of an adult worker. At the time, the basket included limited types of meat, cereals, flours, legumes, vegetables and roots, fruits, as well as cheese and butter, fats, sugars, coffee, tea, milk, and eggs^1^.

Over the years, this composition has changed repeatedly. A recent study investigating taxes and health standards in six Brazilian states identified public policies that included ultra-processed foods such as sausages, margarines, cookies, and ready-to-eat cake dough in the staple food basket^2^.

In response, Decree No. 11,936 was issued on March 5, 2024^3^, outlining new guidelines for composition of the staple food basket by adopting a broader conception of healthy eating, defining it as a “set of foods that seeks to guarantee the human right to adequate and healthy food, and the health and well-being of the Brazilian population”^3^(our translation).

This new perspective transcends the mere supply of sufficient calories to remedy hunger, while also considering the right to regular and permanent access to a diet that meets individual biological and social needs, respecting local and cultural food peculiarities, as well as the dimensions of gender, race, and ethnicity^3,4^. These changes were mainly based on recommendations of the Dietary Guidelines for the Brazilian Population, published in 2014, which emphasizes consumption of unprocessed or minimally processed foods while discouraging the consumption of ultra-processed foods^3^. The Guidelines also values culinary traditions, regional diversities, and sustainable production practices^5^.

Alongside the new decree, Ordinance No 966, of March 6, 2024^6^, was established to specify the foods to be included in the new staple food basket, covering a variety of food groups such as legumes, cereals, roots and tubers, vegetables, fruits, nuts, meat and eggs, milk and cheese, as well as sugars, salt, oils, fats, coffee, tea, yerba mate, and spices^6^.

Public policies for food and nutrition security tend to affect the lowest-income population more intensely, and a crucial principle of these policies lies in the need to prioritize mitigating inequalities without infringing on the autonomy of choice, or disrespecting the food culture or healthy habits already established among the population, especially among those in situations of greater vulnerability.

With the new decree enacted, it is essential to understand to what extent the foods established in the new staple food basket are already purchased by low-income households in Brazil. Thus, this study describes the purchase of foods included in the new staple food basket by low-income households in Brazil, using data from the most recent Household Budget Survey, conducted in 2017–2018.

## METHODS

### Data Source

This study used data from the most recent household food purchase survey of the *Pesquisa de Orçamentos Familiares* (POF - Household Budget Survey) conducted by the *Instituto Brasileiro de Geografia e Estatística* (IBGE - Brazilian Institute of Geography and Statistics) from July 2017 to July 2018^7^.

Its complex sampling plan, structured into two stages, involved the selection of census tracts in the first stage and households in the second. The census tracts were drawn from the IBGE master sample, grouped into household strata with high geographic and socioeconomic homogeneity. Strata construction considered geographic location, place of residence and, within each one, the spectrum of socioeconomic variation according to the head of the household’s income. The estimates obtained are representative of the following domains: Brazil, the five macro-regions (North, Northeast, Midwest, Southeast and South), area (urban or rural), and the 27 Federative Units. A detailed description of the sampling process is available in the IBGE publication^7^.

Study data refer to household food purchases over seven consecutive days, recorded by household residents or by an IBGE interviewer, considering monetary (cash, credit and debit cards) and non-monetary (donation, exchange, or own production) purchases. Data collection was distributed over the four quarters of the year, incorporating the seasonal variety to which household expenditures are subject. The household aggregates generated in the sampling plan (strata) were used as the units of analysis. From 2017 to 2018, the 57,920 households studied were grouped into 575 strata with an average of 86.5 households (ranging from 16 to 524). Socioeconomic and demographic information was also used, collected by the POF through standardized questionnaires^7^.

### Data Organization and Creation of Study Variables

#### Foods from the new staple food basket

Foods included in the new staple food basket were identified in the most recent POF based on Ordinance No. 966 issued by the Ministry of Social Development and Fight Against Hunger (MDS) on March 6, 2024, which defines a list of foods that can make up the new staple food basket^6^.

The new composition includes unprocessed or minimally processed foods (those obtained directly from plants, animals or fungi and acquired for consumption without undergoing any alteration after leaving nature or those subjected to processing that does not add salt, sugars, oils or fats), culinary ingredients (products extracted from unprocessed foods such as oils, fats and sugars, or directly from nature, such as salt), and some processed foods (those manufactured with the addition of salt, sugar, oils or fats to unprocessed or minimally processed foods, such as canned sardines and tuna, cheeses)^6^. The inclusion of ultra-processed foods, defined as industrial formulations typically made with many ingredients and with different stages and types of processing, with little or no presence of unprocessed foods and characterized by the presence of cosmetic food additives or substances for industrial use, is not allowed^6^. This study considered the following ten food groups highlighted in MDS Ordinance No. 966, of March 6, 20246: 1) legumes; 2) cereals; 3) roots and tubers; 4) vegetables; 5) fruits; 6) meat and eggs; 7) milk and cheese; 8) culinary ingredients (identified in the Ordinance as “sugars, salt, oils and fats”); 9) nuts and oilseeds; and 10) coffee, tea, yerba mate and spices. For a more detailed analysis, we created food subgroups, except for the group of nuts and walnuts. Box presents an extensive list of food examples from each group.

**Box:** Food groupsa and subgroups in the new staple food basket.

Groups	Subgroups	Food examples
Cereals	Rice, corn, and other grains	White, brown, or parboiled rice, in bulk or packaged; corn kernels or on the cob, wheat grains, oats
	Breads	Breads made only from wheat flour and/or other flours made from unprocessed and minimally processed foods, yeast, water, salt
	Corn, wheat, and other cereal flours	Corn, wheat, rice, oatmeal, linseed, sorghum flour
	Pasta	Fresh or dry pasta or noodles made with these flours/semolina, water and/or eggs, and/or other unprocessed or minimally processed foods
Culinary ingredients	Vegetable oils	Soybean, sunflower, maize, palm oils, among vegetable oils; olive oil
	Sugars	White, demerara or brown table sugar and honey
	Butter	With or without salt
	Lard	Lard
Meat and eggs	Poultry meat	Chicken and other poultry
	Beef	Beef
	Eggs	Eggs of chicken and other poultry
	Pork	Pork
	Fish	All fish commonly consumed
	Canned sardines and tuna	Canned sardines or tuna
	Sheep and goat meat	Lamb, mutton, goat
	Other unprocessed or minimally processed meats	Offal and other parts and meats rarely consumed
Milk and cheeses	Milk	Pasteurized or industrialized milk in the form of ultra-pasteurized, powdered, whole, semi-skimmed or skimmed
	Cheese	Cheeses made from milk and salt and microorganisms used for fermentation
	Natural yogurt	Natural yogurt without added sugar, sweetener and/or additives that modify the sensory characteristics of the product
Legumes	Beans	Beans of all colors (black, white, purple, mulatinho, green, pinto, cowpea, kidney, butter, jalo, andú, among others)
	Other legumes	Peas, lentils, chickpeas, broad beans, pigeon pea, snappy pea
Roots and tubers	Cassava flour and from other roots and tubers	Minimally processed cassava flours, among other flours
	Cassava, potatoes and other roots and tubers	*Ária*, sweet potato, potato, arracacha, crem potato, potato yam, amazonian yam, yam, cassava, and other roots and tubers unprocessed or packaged, fractionated, refrigerated or frozen
	Preparations derived from cassava	Carimã flour, uarini flour; maniçoba and tucupi, tapioca flour/starch, among others
Fruits	Unprocessed or dried	Unprocessed fruit or dried fruit packaged, fractionated, refrigerated or frozen. Examples: avocado, pineapple, *abiu, abricó*, açaí, *açaí-solteiro, acerola*, plum, blackberry, *araçá, araçá-boi, araçá-pera, araticum, aroeira-pimenteira, arumbeva, atemoia, babaçu, bacaba, bacupari, bacuri*, banana, *baru, biribá, brejaúva, buriti, butiá*, cocoa, *cagaita, cajarana, cajá*, cashew, *caju do cerrado, cajuí, cambuci, cambuí, camu-camu*, persimmon, *carambola, cereja-do-rio-grande, ciriguela*, coconut, *coco-cabeçudo, coco-indaiá, coquinho-azedo, coroa-de-frade, croá, cubiu, cupuaçu, cupuí, cutite, curriola*, fig, *fisalis, fruta-pão*, guava, *goiaba-serrana*, soursop, *guabiroba, grumixama, guapeva, guaraná, inajá, ingá*, jackfruit, *jabuticaba, jambo, jambolão, jaracatiá, jatobá, jenipapo, juá, juçara, jurubeba*, kiwi, orange, lemon, *lobeira*, apple, *macaúba, mama-cadela*, papaya, *mandacaru*, mango, *mangaba, mapati*, passion fruit, *marmelada-de-cachorro*, watermelon, melon, tangerine, strawberry, *murici*, nectarine, *pajurá, patauá, pequi*, pear, *pera-do-cerrado*, peach, *piquiá*, sugar-apple, pine nut, *pitanga, pitomba, pupunha*, pomegranate, *sapucaia, sapoti, sapota, seriguela, sete-capotes, sorva, tamarindo, taperebá, tucumã, umari, umbu, umbu-cajá*, grape, *uvaia, uxi, xixá*, among others
	Pulp	Fruit pulps without added sugar
Vegetables	Unprocessed	Vegetables and greens unprocessed or packaged, fractionated, refrigerated or frozen, such as pumpkin, zucchini, chard, watercress, lettuce, chicory, garlic, leek, sorrel, eggplant, beetroot, purslane, *bertalha*, broccoli, bamboo shoots, *capicoba*, nasturtium, needlebur, *caruru, catalonha*, onion, chives, carrot, parsley, *chicória-paraense*, chayote, wild cabbage, cauliflower, *croá, crem*, dandelion, escarole, spinach, *gueroba, gila, guariroba, jambu*, scarlet eggplant, *jurubeba, major-gomes, maxixe, mini-pepininho*, mustard, *muricato*, *ora-pro-nóbis*, palm, cucumber, *peperômia*, bell pepper, *puxuri*, okra, radish, cabbage, arugula, parsley, sow thistle, *taioba*, tomato, nettle, sorrel, green beans, among others
	Preserves and extracts	Carrots, cucumbers, hearts of palm, onions, cauliflower, and other vegetables preserved in brine or in a salt and vinegar solution; tomato extract or concentrates and/or other unprocessed and minimally processed foods (with salt and/or sugar)^a^
Coffee, tea, yerba mate and spices	Spices	Pepper, black pepper, cinnamon, cumin, cloves, coriander, nutmeg, ginger, saffron, turmeric, among others
	Coffee	Ground coffee, coffee beans
	Yerba mate	unprocessed yerba mate
	Tea	Chamomile, fennel, cinnamon, among other teas
Chestnuts and walnuts	-	Peanuts, cashew nuts, baru nuts, Brazil nuts, cutia nuts, egg nuts, chichá nuts, licuri nuts, macaúba nuts, and other oilseeds without salt or sugar

^a^ Ordinance of the Ministry of Social Development and Fight Against Hunger No. 966, of March 6, 2024.

Source: Prepared based on Ordinance No. 966/2024.

Correction factors were applied to exclude the inedible fraction of the foods^8^. Subsequently, the quantities of food purchased (kilograms or liters) were converted into calories using the Brazilian Food Composition Table, developed by the Universidade de São Paulo^9^.

## Socioeconomic and Demographic Variables

The socioeconomic and demographic variables used in this study were: gender (male and female), race/skin color self-declared by the head of the household (White, Brown/Mixed-race [*pardo*], Black, Yellow, Indigenous), education of the head of the household (illiterate, incomplete primary, complete primary, incomplete secondary, complete secondary, or incomplete tertiary or higher), presence of children up to 5 years old in the household (yes or no), receives *Bolsa Família* (yes or no), place of residence (urban and rural), and Major Regions (North, Northeast, Midwest, Southeast and South), in addition to the average monthly per capita family income.

The monthly per capita household income was estimated as the sum of the total gross monetary income of the components of the consumption units in a month divided by the number of household residents. Total income was obtained from monetary and non-monetary income information, including the receipt of *Bolsa Família* or other income transfer programs.

## Data Analysis

Only POF households in low-income situations were included in the study, defined as those with a monthly per capita family income of up to half the minimum wage^10^, using the 2018 monthly minimum wage as a reference, equivalent to 954 reais (R$)^11^, corresponding to R$ 477 for half a wage. A total of 13,706 households were analyzed (23.66% of the total POF sample).

Firstly, the socioeconomic and demographic characteristics of the households were described, presenting the proportions (%) with their respective 95% confidence intervals (95%CI).

Next, the share of purchased food groups and subgroups from the new staple food basket was estimated. Estimates were described based on the mean percentage of total calories (% kcal), with their respective 95% confidence intervals, at a national level and according to the place of residence (urban and rural), the major regions of the country and the race/skin color self-declared by the head of the household.

In the analyses by race/skin color, only households headed by Black, brown, and White individuals were evaluated. Yellow and Indigenous heads of household were not included due to their small sample size (less than 1% of the total households). Households headed by Black and Brown people were evaluated separately and in a combined category (Black population).

Finally, weighting factors were applied considering the sampling structure, allowing the extrapolation of results to the entire Brazilian population. All analyses were performed on Stata 18.0/SE and the results were reviewed by all authors.

## RESULTS

In Brazil, in 2017–2018, most heads of households in low-income situations were men (59.0%), self-declared Brown (54.4%) and had incomplete primary education (36.0%). Most of the households received *Bolsa Família* (60.2%) and more than a third (36.4%) had children up to five years of age. The households were predominantly located in the urban area (73.2%) and in the Northeast region (47.4%) (Table 1).

**Table 1:** Socioeconomic characteristics of low-income households. Brazil, 2017–2018.

Socioeconomic characteristics	%	95%CI
Gender of the head of the household		
Man	59.0	55.9–64.0
Woman	41.0	36.1–44.1
Race/skin color self-declared by the head of the household		
Brown/Mixed-race	54.4	48.4–60.2
White	34.3	28.8–40.2
Black	10.4	7.4–14.5
Yellow	0.5	0.0–1.0
Indigenous	0.4	0.1–0.9
Education of the head of the household		
Illiterate/no formal education	12.9	9.8–16.1
Incomplete Primary Education	36.0	30.4–41.5
Complete Primary Education	8.7	6.1–11.3
Incomplete Secondary Education	5.9	4.2–7.6
Complete Secondary Education	23.0	21.3–24.7
Incomplete Tertiary Education	11.6	9.5–13.7
Children up to 5 years old in the household		
Yes	36.4	30.5–42.3
No	63.6	57.7–69.5
Bolsa Família		
Yes	60.2	54.3–65.9
No	39.8	34.1–45.7
Household setting		
Urban	73.2	67.8–78.0
Rural	26.8	22.0–32.2
Region		
Northeast	47.4	41.5–53.3
Southeast	26.3	21.2–32.1
North	13.8	9.8–19.1
South	7.1	5.2–9.6
Midwest	5.3	3.9–7.5

95%CI: 95% confidence interval.

Table 2 describes the average calorie share (%) of the food groups and subgroups that make up the new staple food basket in low-income households nationally and according to place of residence. The foods included in the new staple basket represented 84.1% of the total calories purchased by low-income households in Brazil, being higher in rural areas (88.1%) compared to urban areas (82.7%). Cereals (33.5%), culinary ingredients (21.3%), and meat and eggs (11.6%), followed by milk and cheese (5.1%) and legumes (5.0%), made the greatest contributions at the national level. In the cereal group, the rice, corn, and other grains subgroup contributed 19.7%, whereas beans, from the legumes group, represented 4.7% of calories. Regarding the culinary ingredients group, vegetable oils and sugars contributed approximately 10.3% and 10.6%, respectively. In the meat and egg group, the most consumed meats were poultry and beef, with 5.0% and 4.0% of calories, respectively. Fruits and vegetables accounted for 1.9% and 0.8% of calories, respectively.

**Table 2:** Average calorie share (%) from food groups and subgroups that make up the new staple food basket in low-income households nationally and according to place of residence. Brazil, 2017–2018.

Food groups and subgroups in the new staple food basket	Average calorie share (%)					
	Brazil		Urban area		Rural area	
	%	95%CI	%	95%CI	%	95%CI
**Cereals**	**33.5**	**32.4–34.6**	**33.3**	**32.1–34.5**	**34.0**	**31.0–36.6**
Rice, corn, and other grains	19.7	18.4–21.0	18.5	17.1–20.0	22.7	20.1–25.4
Breads^a^	8.2	7.5–8.8	9.6	8.9–10.32	4.3	3.7–4.9
Corn, wheat, and other cereal flours	3.0	2.7–3.3	2.5	2.2–2.8	4.4	3.5–5.4
Pasta^b^	2.7	2.5–2.9	2.7	2.5–3.0	2.6	2.2–3.0
**Culinary ingredients**	**21.3**	**20.5–22.1**	**20.6**	**19.6–21.5**	**23.3**	**22.2–24.5**
Vegetable oils	10.3	9.8–10.9	10.1	9.4–10.9	10.9	10.1–11.7
Sugars	10.6	10.0–11.1	10.0	9.3–10.7	12.1	11.3–12.9
Butter	0.3	0.2–0.4	0.3	0.2–0.4	0.2	0.1–0.3
Lard	0.1	0.0–0.2	0.1	0.0–0.2	0.1	0.0–0.3
**Meat and eggs**	**11.6**	**11.0–12.1**	**11.3**	**10.7–11.9**	**12.3**	**11.5–13.2**
Poultry meat	5.0	4.7–5.3	4.8	4.4–5.2	5.4	4.9–5.9
Beef	4.0	3.8–4.3	4.1	3.8–4.4	3.9	3.4–4.3
Eggs	0.9	0.7–0.9	0.9	0.8–1.0	0.9	0.7–1.1
Pork	0.8	0.8–1.0	0.8	0.6–0.9	0.8	0.6–1.0
Fish	0.7	0.5–0.8	0.5	0.4–0.6	1.1	0.7–1.4
Canned sardines and tuna	0.1	0.1–0.1	0.1	0.1–0.1	0.1	0.1–0.1
Sheep and goat meat	0.1	0.1–0.1	0.1	0.0–0.1	0.2	0.1–0.3
Other unprocessed or minimally processed meats	0.0	0.0–0.1	0.0	0.0–0.1	0.0	0.0–0.1
**Milk and cheeses**	**5.1**	**4.8–5.4**	**5.4**	**5.0–5.8**	**4.4**	**4.7–5.0**
Milk	4.6	4.3–4.9	4.7	4.4–5.1	4.1	3.5–4.7
Cheese^c^	0.5	0.4–0.6	0.6	0.5–0.7	0.3	0.2–0.3
Natural yogurt^d^	0.0	0.0–0–0	0.0	0.0–0.0	0.0	0.0–0.0
**Legumes**	**5.0**	**4.7–5.3**	**4.9**	**4.6–5.3**	**5.3**	**4.6–5.9**
Beans	4.7	4.4–5.0	4.6	4.3–4.9	4.9	4.3–5.5
Other legumes	0.4	0.3–0.5	0.4	0.2–0.5	0.4	0.2–0.6
**Roots and tubers**	**4.8**	**4.0–5.5**	**4.2**	**3.6–4.8**	**6.4**	**4.3–8.4**
Cassava flour and from other roots and tubers	3.2	2.5–4.0	2.7	2.1–3.3	4.8	2.6–6.9
Cassava, potatoes and other roots and tubers	0.9	0.8–1.0	0.9	0.8–1.0	0.8	0.7–1.0
Preparations derived from cassava^e^	0.6	0.5–0.7	0.6	0.5–0.7	0.7	0.5–1.0
**Fruits**	**1.9**	**1.8–2.1**	**2.0**	**1.8–2.2**	**1.6**	**1.3–1.9**
Unprocessed or dried	1.9	1.7–2.1	2.0	1.8–2.2	1.6	1.3–1.9
Pulp^f^	0.0	0.0–0.0	0.0	0.0–0.0	0.0	0.0–0.1
**Vegetables**	**0.8**	**0.7–0.8**	**0.8**	**0.7–0.9**	**0.6**	**0.5–0.7**
Unprocessed	0.7	0.7–0.8	0.8	0.7–0.9	0.6	0.5–0.7
Preserves and extracts	0.0	0.0–0.0	0.0	0.0–0.0	0.0	0.0–0.0
**Coffee, tea, yerba mate, and spices**	**0.2**	**0.1–0.2**	**0.2**	**0.1–0.2**	**0.2**	**0.1–0.2**
Spices^g^	0.1	0.1–0.1	0.1	0.1–0–1	0.1	0.1–0.2
Coffee	0.1	0.0–0.1	0.1	0.1–0.1	0.1	0.1–0.1
Yerba mate	0.0	0.0–0.0	0.0	0.0–0.0	0.0	0.0–0.0
Tea	0.0	0.0–0.0	0.0	0.0–0.0	0.0	0.0–00
**Nuts and oilseeds** ^h^	**0.0**	**0.0–0.0**	**0.0**	**0.0–0.0**	**0.0**	**0.0–0.0**
Total	84.1	83.4–84.9	82.7	81.8–83.6	88.1	87.2–88.9

95%CI: 95% confidence interval.

^a^ Made only from wheat flour and/or other flours made from unprocessed and minimally processed foods, yeasts, water, salt and/or other unprocessed and minimally processed.

^b^ Unprocessed or dried pasta made with flour/semolina, water and/or eggs and/or other unprocessed or minimally processed foods.

^c^ Made from milk and salt and microorganisms used for fermentation.

^d^ Without added sugar, sweetener and/or additives that modify the sensory characteristics of the product.

^e^ Maniçoba, tucupi and tapioca flour/starch, among others.

^f^ No added sugar.

^g^ Pepper, nutmeg, saffron, among others.

^h^ No salt or sugar.

Households in rural areas had higher caloric intakes, compared to urban areas, of rice, corn and other grains (22.7% *versus* 18.5%), corn, wheat and other cereal flours (4.4% *versus* 2.5%), sugars (12.1% *versus* 10.0%), fish (1.1% *versus* 0.5%), and cassava or other roots and tubers flours (4.8% *versus* 2.7%). On the other hand, households in urban areas had, in relation to rural areas, a greater share of bread (9.6% *versus* 4.3%), milk (4.7% *versus* 4.1%), cheese (0.6% *versus* 0.3%), fruits (2.0% *versus* 1.6%), and vegetables (0.8% *versus* 0.6%).

Table 3 shows the average calorie share (%) of the food groups and subgroups in the new staple food basket in low-income households according to the major regions of the country. Food share was higher in the North (88.2%) and Northeast (85.9%) regions, intermediate in the Midwest (82.8%), and lower in the Southeast (80.4%) and South (79.2%). Northern Brazil stood out, in relation to other regions, for its high contribution of cassava and other roots and tubers flours (10.2%), poultry meat (6.8%), and fish (2.1%). The South stood out for the greater caloric contribution of corn, wheat and other cereals (7.3%), the Southeast for milk (6.1%), and the Midwest for rice, corn, and other grains (24.3%). Beans had the highest caloric contribution in the Northeast (5.4%) and the lowest, in the South (3.0%). The caloric share of fruits varied from 2.3% in the North to 1.7% in the Northeast, whereas the share of vegetables and greens varied from 0.9% in the Midwest to 0.4% in the North.

**Table 3:** Average calorie share (%) of the food groups and subgroups in the new staple food basket in low-income households according to the major regions of the country. Brazil, 2017–2018.

Food groups and subgroups in the new staple food basket	Average calorie share (%)									
	North		Northeast		Southeast		South		Midwest	
	%	95%CI	%	95%CI	%	95%CI	%	95%CI	%	95%CI
**Cereals**	**29.4**	**25.4–33.4**	**35.8**	**34.3–37.2**	**32.2**	**30.2–34.1**	**30.1**	**27.9–32.3**	**34.8**	**30.9–38.8**
Rice, corn, and other grains	18.9	14.9–22.9	20.7	18.7–22.8	18.5	16.6–20.3	15.2	13.1–17.3	24.3	20.6–27.9
Breads^a^	6.8	4.8–8.8	8.6	7.8–9.4	8.9	7.5–10.3	5.2	4.0–6.5	7.5	3.9–11.0
Corn, wheat, and other cereal flours	1.8	1.4–2.3	3.4	2.8–4.0	2.0	1.6–2.4	7.3	5.6–9.0	1.7	1.0–2.4
Pasta^b^	1.8	1.6–2.1	3.0	2.7–3.4	2.8	2.3–3.2	2.4	1.9–2.9	1.4	1.2–1.7
**Culinary ingredients**	**20.7**	**19.1–22.3**	**19.9**	**19.1–20.8**	**23.3**	**21.3–25.3**	**23.8**	**21.0–26.6**	**21.8**	**18.7–24.9**
Vegetable oils	10.6	9.1–12.0	8.8	8.1–9.3	12.1	10.7–13.5	12.9	10.4–15.3	11.9	10.1–13.7
Sugars	9.8	8.8–10.8	10.9	10.3–11.42	10.8	9.2–12.5	10.3	8.5–12.0	9.6	7.5–11.7
Butter	0.3	0.2–0.5	0.3	0.3–0.4	0.2	0.1–0.4	0.1	0.0–0.1	0.2	0.0–0.3
Lard	0.0	0.0–0.0	0.0	0.0–0.0	0.2	0.0–0.4	0.6	0.2–1.0	0.1	0.0–0.2
**Meat and eggs**	**16.0**	**14.8–17.1**	**12.1**	**11.6–12.7**	**8.3**	**7.5–9.1**	**11.1**	**9.5–12.6**	**11.9**	**10.4–13.4**
Poultry meat	6.8	6.1–7.6	5.7	5.4–6.1	3.0	2.6–3.5	4.1	3.3–4.9	3.8	3.1–4.5
Beef	5.4	4.7–6.1	3.9	3.6–4.2	3.0	2.6–3.5	4.6	3.6–5.5	5.9	4.8–7.0
Eggs	0.6	0.5–0.7	1.0	0.9–1.1	0.9	0.7–1.0	0.8	0.6–1.0	0.7	0.6–0.8
Pork	0.3	0.2–0.5	0.6	0.4–0.7	1.1	0.9–1.4	1.5	1.0–2.0	1.2	0.5–2.0
Fish	2.2	1.6–2.7	0.6	0.5–0.8	0.2	0.1–0.3	0.0	0.0–0.1	0.1	0.0–0.3
Canned sardines and tuna	0.1	0.0–0.1	0.1	0.1–0.1	0.0	0.0–0.1	0.0	0.0–0.1	0.1	0.0–0.1
Sheep and goat meat	0.2	0.1–0.3	0.1	0.1–0.2	0.0	0.0–0.0	0.0	0.0–0.1	0.1	0.0–0.2
Other unprocessed or minimally processed meats	0.3	0.1–0.6	0.0	0.0–0.0	-	-	-	-	-	-
**Milk and cheeses**	**3.9**	**3.2–4.5**	**4.4**	**4.1–4.7**	**6.8**	**6.0–7.6**	**6.2**	**5.4–7.0**	**4.5**	**3.7–5.3**
Milk	3.6	3.0–4.2	3.9	3.6–4.2	6.1	5.3–7.0	5.4	4.7–6.2	4.0	3.2–4.8
Cheese^c^	0.2	0.1–0.3	0.5	0.4–0.6	0.7	0.5–0.8	0.7	0.5–0.9	0.5	0.3–0.6
Natural yogurt^d^	0.0	0.0–0.0	0.0	0.0–0.0	0.0	0.0–0.0	0.0	0.0–0.0	0.0	0.0–0.0
**Legumes**	**3.6**	**3.3–4.0**	**5.9**	**5.5–6.3**	**4.9**	**4.2–5.6**	**3.6**	**2.9–4.3**	**4.1**	**3.2–4.9**
Beans	3.4	3.0–3.7	5.4	4.9–5.9	4.6	5.0–5.8	3.0	2.3–3.7	3.8	2.9–4.6
Other legumes	0.3	0.2–0.4	0.4	0.3–0.6	0.3	0.1–0.4	0.5	0.0–1.0	0.3	0.1–0.5
**Roots and tubers**	**11.6**	**8.5–14.7**	**5.1**	**4.6–5.6**	**2.0**	**1.6–2.3**	**1.7**	**1.3–2.0**	**2.4**	**1.6–3.1**
Cassava flour and from other roots and tubers	10.2	6.9–13.6	3.4	2.9–3.9	0.7	0.5–0.9	0.2	0.1–0.4	0.6	0.3–0.8
Cassava, potatoes, and other roots and tubers	0.5	0.3–0.6	0.8	0.7–1.0	1.0	0.9–1.2	1.3	1.0–1.5	1.3	0.8–1.8
Preparations derived from cassava^e^	0.9	0.6–1.2	0.9	0.7–1.1	0.2	0.1–0.3	0.2	0.1–0.3	0.5	0.1–0.9
**Fruits**	**2.4**	**1.7–3.0**	**1.8**	**1.6–1.9**	**1.9**	**1.6–2.3**	**1.8**	**1.4–2.1**	**2.3**	**1.7–2.9**
Unprocessed or dried	2.3	1.6–3.0	1.7	1.6–1.9	1.9	1.6–2.3	1.8	1.4–2.1	2.3	1.7–3.0
Pulp^f^	0.1	0.0–0.1	0.0	0.0–0.0	0.0	0.0–0.0	-	-	0.0	0.0–0.0
**Vegetables**	**0.4**	**0.4–0.5**	**0.8**	**0.7–0.8**	**0.9**	**0.8–1.0**	**0.8**	**0.7–1.0**	**1.0**	**0.7–1.2**
Unprocessed	0.4	0.4–0.5	0.7	0.7–0.8	0.9	0.7–1.0	0.8	0.6–0.9	0.9	0.7–1.2
Preserves and extracts	0.0	0.0–0.0	0.0	0.0–0.0	0.0	0.0–0.0	0.0	0.0–0.1	0.0	0.0–0.0
**Coffee, tea, yerba mate, and spices**	**0.2**	**0.1–0.2**	**0.2**	**0.2–0.2**	**0.1**	**0.1–0.2**	**0.1**	**0.1–0.2**	**0.1**	**0.1–0.2**
Spices^g^	0.1	0.1–0.2	0.1	0.1–0.2	0.1	0.0–0.1	0.1	0.0–0.1	0.1	0.1–0.1
Coffee	0.1	0.0–0.1	0.1	0.1–0.2	0.1	0.0–0.1	0.1	0.0–0.1	0.1	0.0–0.1
Yerba mate	-	-	-	-	0.0	0.0–0.0	0.0	0.0–0.0	0.0	0.0–0.1
Tea	0.0	0.0–0.0	0.0	0.0–0.0	0.0	0.0–0.0	0.0	0.0–0.0	0.0	0.0–0.0
**Nuts and oilseeds** ^h^	**0.0**	**0.0–0.1**	**0.0**	**0.0–0.0**	**0.0**	**0.0–0.0**	**0.0**	**0.0–0.1**	**0.0**	**0.0–0.1**
Total	88.2	87.4–89.1	85.9	85.0–86.8	80.4	78.7–82.1	79.2	77.1–81.2	82.8	80.4–85.3

95%CI: 95% confidence interval.

^a^ Made only from wheat flour and/or other flours made from unprocessed and minimally processed foods, yeasts, water, salt and/or other unprocessed and minimally processed.

^b^ Unprocessed or dried pasta made with flour/semolina, water and/or eggs and/or other unprocessed or minimally processed foods.

^c^ Made from milk and salt and microorganisms used for fermentation.

^d^ Without added sugar, sweetener and/or additives that modify the sensory characteristics of the product.

^e^ Maniçoba, tucupi and tapioca flour/starch, among others.

^f^ No added sugar.

^g^ Pepper, nutmeg, saffron, among others.

^h^ No salt or sugar.

Table 4 presents the average calorie participation (%) of the food groups and subgroups in the new staple food basket in low-income households according to the head of the household’s self-declared race/skin color. Few differences was observed in food share between the race/skin color categories, ranging from 85.2% of calories among Brown to 82.5% among White individuals. The caloric contribution of meat and eggs was slightly higher in households headed by Brown individuals (12.1%) compared to that observed in households headed by White individuals (10.5%).

**Table 4:** Average calorie participation (%) of the food groups and subgroups of the new staple food basket in low-income households according to the self-declared* race/skin color of the head of the household. Brazil, 2017–2018.

Food groups and subcategories in the new staple food basket	Average calorie share (%)							
	White		Black Population**		Black		Brown/Mixed-race	
	%	95%CI	%	95%CI	%	95%CI	%	95%CI
**Cereals**	**32.6**	**30.5–34.7**	**34.0**	**32.7–35.5**	**32.1**	**29.6–34.6**	**34.4**	**32.9–35.9**
Rice, corn, and other grains	18.8	16.8–20.8	20.2	18.6–21.8	18.7	16.5–20.9	20.5	18.6–22.3
Breads^a^	7.7	6.8–8.7	8.3	7.5–9.2	7.7	6.0–9.3	8.5	7.5–9.4
Corn, wheat, and other cereal flours	3.4	2.7–4.0	2.8	2.4–3.2	2.6	1.5–3.7	2.9	2.4–3.3
Pasta^b^	2.7	2.4–3.0	2.7	2.4–2.9	3.1	2.4–3.8	2.6	2.3–2.8
**Culinary ingredients**	**22.0**	**20.5–23.5**	**21.0**	**20.1–21.8**	**20.6**	**18.7–22.4**	**21.0**	**20.1–22.0**
Vegetable oils	10.9	9.9–11.9	10.0	9.4–10.7	10.0	8.3–11.6	10.1	9.3–10.8
Sugars	10.7	9.6–11.8	10.5	9.9–11.1	10.1	8.6–11.5	10.6	9.9–11.3
Butter	0.2	0.1–0.3	0.3	0.2–0.4	0.4	0.1–0.6	0.3	0.2–0.4
Lard	0.2	0.0–0.4	0.1	0.0–0.1	0.2	0.1–0.3	0.0	0.0–0.0
**Meat and eggs**	**10.5**	**9.7–11.3**	**12.1**	**11.4–12.8**	**11.5**	**10.0–12.9**	**12.2**	**11.5–13.0**
Poultry meat	4.4	3.8–4.9	5.3	4.9–5.6	4.9	4.1–5.7	5.4	5.0–5.8
Beef	3.7	3.3–4.1	4.2	3.9–4.5	4.4	3.7–5.1	4.2	3.8–4.5
Eggs	0.9	0.8–1.0	0.7	0.8–1.0	1.0	0.7–1.3	0.9	0.6–1.2
Pork	0.9	0.7–1.2	0.9	0.6–0.8	0.5	0.2–0.7	0.7	0.6–0.8
Fish	0.4	0.2–0.6	0.8	0.6–1.0	0.5	0.3–0.7	0.9	0.7–1.1
Canned sardines and tuna	0.1	0.0–0.1	0.1	0.0–0.1	0.1	0.0–0.1	0.1	0.0–0.1
Sheep and goat meat	0.1	0.0–0.1	0.1	0.0–0.1	0.1	0.0–0.2	0.1	0.0–0.1
Other unprocessed or minimally processed meats	0.0	0.0–0.0	0.1	0.0–0.1	0.1	0.0–0.2	0.1	0.0–0.1
**Milk and cheeses**	**5.6**	**4.9–6.2**	**4.8**	**4.4–5.2**	**5.8**	**4.3–7.3**	**4.6**	**4.2–5.0**
Milk	5.9	4.3–5.5	4.3	4.0–4.7	5.3	3.9–6.6	4.2	3.8–4.5
Cheese^c^	0.7	0.5–0.8	0.5	0.4–0.5	0.5	0.3–0.7	0.4	0.4–0.5
Natural yogurt^d^	0.0	0.0–0.0	0.0	0.0–0.0	0.0	0.0–0.0	0.0	0.0–0.0
**Legumes**	**4.8**	**4.3–5.3**	**5.2**	**4.8–5.5**	**5.7**	**4.8–6.7**	**5.1**	**4.7–5.4**
Beans	4.5	4.0–5.0	4.8	4.4–5.1	5.4	4.5–6.3	4.6	4.3–5.0
Other legumes	0.3	0.2–0.5	0.4	0.3–0.5	0.3	0.1–0.4	0.4	0.3–0.5
**Roots and tubers**	**4.2**	**2.4–5.9**	**5.1**	**4.4–5.8**	**5.1**	**3.5–6.8**	**5.1**	**4.3–5.8**
Cassava flour and from other roots and tubers	2.7	0.8–4.5	3.5	2.8–4.2	3.7	1.9–5.4	3.5	2.8–4.2
Cassava, potatoes and other roots and tubers	1.0	0.9–1.2	0.8	0.7–0.9	0.9	0.6–1.1	0.8	0.7–0.9
Preparations derived from cassava^e^	0.5	0.3–0.6	0.7	0.6–0.9	0.6	0.2–0.9	0.8	0.8–2.2
**Fruits**	**1.9**	**1.6–2.1**	**1.9**	**1.7–2.2**	**2.0**	**1.4–2.6**	**1.9**	**1.7–2.2**
Unprocessed or dried	1.8	1.6–2.1	1.9	1.7–2.1	2.0	1.4–2.6	1.9	1.7–2.1
Pulp^f^	0.0	0.0–0.0	0.0	0.0–0.0	0.0	0.0–0.0	0.0	0.0–0.0
**Vegetables**	**0.8**	**0.7–0.9**	**0.7**	**0.7–0.8**	**0.8**	**0.7–0.9**	**0.7**	**0.7–0.8**
Unprocessed	0.8	0.7–0.9	0.7	0.6–0.8	0.8	0.6–0.9	0.7	0.6–0.8
Preserves and extracts	0.0	0.0–0.0	0.0	0.0–0.0	0.0	0.0–0.0	0.0	0.0–0.0
**Coffee, tea, yerba mate, and spices**	**0.2**	**0.1–0.2**	**0.2**	**0.1–0.2**	**0.2**	**0.1–0.3**	**0.2**	**0.1–0.2**
Spices^g^	0.1	0.0–0.1	0.1	0.0–0.1	0.1	0.1–0.2	0.1	0.1–0.1
Coffee	0.1	0.0–0.1	0.1	0.0–0.1	0.1	0.0–0.1	0.1	0.0–0.1
Yerba mate	0.0	0.0–0.0	0.0	0.0–0.0	0.0	0.0–0.0	0.0	0.0–0.0
Tea	0.0	0.0–0.0	0.0	0.0–0.0	0.0	0.0–0.0	0.0	0.0–0.0
**Nuts and oilseeds** ^h^	**0.0**	**0.0–0.0**	**0.0**	**0.0–0.0**	**0.0**	**0.0–0.0**	**0.0**	**0.0–0.0**
Total	82.5	81.1–83.8	85.0	84.1–85.9	83.8	81.6–85.9	85.2	84.3–86.2

95%CI: 95% confidence interval.

*Only households headed by Black, Brown, and White people were evaluated. Yellow and Indigenous households were not included due to their low sample size.

**Black and Brown grouped.

^a^ Made only from wheat flour and/or other flours made from unprocessed and minimally processed foods, yeasts, water, salt and/or other unprocessed and minimally processed.

^b^ Unprocessed or dried pasta made with flour/semolina, water and/or eggs and/or other unprocessed or minimally processed foods.

^c^ Made from milk and salt and microorganisms used for fermentation.

^d^ Without added sugar, sweetener and/or additives that modify the sensory characteristics of the product.

^e^ Maniçoba, tucupi and tapioca flour/starch, among others.

^f^ No added sugar.

^g^ Pepper, nutmeg, saffron, among others.

^h^ No salt or sugar.

Estimates of the average caloric intake of foods in the New Staple Basket, detailed for Brazil and for major regions, can be accessed in a public online repository^a^.

## DISCUSSION

In 2017–2018, the foods included in the new staple food basket formed the basis of diet in low-income households across all sociodemographic strata evaluated, representing almost 85.0% of total calories consumed. This value exceeded 88.0% in rural households and those in Northern region of Brazil.

Our findings are significant because they indicate that the new staple food basket, in addition to being healthy and sustainable, already reflects a dietary pattern widely consumed by the Brazilian population. This reinforces the cultural viability of implementing the basket in public policies and challenges hegemonic and misguided discourses, often based on the common assumption that ultra-processed foods would be important food sources for populations at greater risk of food insecurity^12,13^.

In this regard, the high consumption of meat and eggs by this population stands out by exceeding 11% of total calories, similar to that of the general population (10.8% of calories)^14^. Importantly, this percentage refers to animal-source proteins, exclusing those of plant origin (such as beans), indicating that, on their own, this group already meets adequate parameters of international protein recommendations^12^ by the low-income population. However, this is not always positive since excessive consumption of meat, although an important source of nutrients, is associated with an increased risk of certain cancers and cardiovascular diseases^15^ and has a high environmental impact^18^. In Brazil, the increasing share of beef in the diet has been consistent in recent decades, especially in the lower-income groups^14^.

On the other hand, these groups showed insufficient consumption of fruits and vegetables. These foods, whose adequate consumption prevents excessive weight gain and reduces the risk of several chronic non-communicable diseases (NCDs). Yet, they accounted for only 2.7% of total calories, less than half of the amount recommended by the World Health Organization (at least 400 grams daily or about 5-6% of the total calories of a 2,000 kcal diet)^21^, a value below the national average (3.7% of calories)^14^. Sugar consumption, in turn, was excessive, exceeding 10% of calories in all subgroups evaluated, except for the Northeast region. High sugar intake is a public health concern, as it has been associated with a worsening of diet nutritional quality, excessive energy consumption, and the risk of developing obesity and NCDs^22,23^.

Our results, which showed small differences in race/skin color, corroborate a previous study that evaluated the food consumption of the Brazilian population in 2017–2018^24^. This study revealed that Black and Brown people showed only slightly greater adherence to the golden rule of the Dietary Guidelines for the Brazilian Population. However, this did not necessarily translate into higher overall diet quality, since this difference was mainly explained by higher consumption of basic foods such as rice and beans, whereas the same did not occur for other natural or minimally processed foods such as fruits, vegetables, nuts and oilseeds^24^. Moreover, these differences in food consumption were largely explained by differences in socioeconomic conditions between race/skin color strata^24^, and it is likely that by evaluating only low-income people in the present study, this difference was further attenuated.

In Brazil, adherence to a dietary pattern based on unprocessed or minimally processed foods is higher among populations from lower socioeconomic strata^25^. However, a recent study found an increase in the consumption of ultra-processed foods, from 2008 to 2018, among the Brazilian population, particularly higher among poorer, less educated individuals, residents of rural areas, as well as those living in the North and Northeast regions, and Black and Brown people.^25^. This indicates a national standardization at higher levels and a concentration of risks among the most vulnerable populations.

These changes in the new staple food basket therefore may positively impact the population’s health, since a dietary pattern based on unprocessed or minimally processed foods is associated with greater nutritional quality and a lower risk of various NCDs, such as obesity, diabetes, and cardiovascular diseases^26,27^. Moreover, these foods, when of plant origin, have positive effects on environmental sustainability, producing a lower carbon and water footprint and fostering agrobiodiversity^28^. This is even more relevant when we consider that NCDs and climate change affect the poorest and most peripheral populations more intensely^28^.

Regional and urban-rural differences were observed in food groups and subgroups, indicating dietary identity and how food consumption varies in Brazil. This corroborates the finding of Levy et al.^14^ (2023), whose data revealed a difference in food consumption among different strata. Moreover, the new basket also encourages the consumption of regional foods and sociobiodiversity which are important for culture, the local economy, and the environment^5,29^. This is crucial considering that regional foods represented only 3.12% of the caloric share in 2017–2018, exhibiting a significant decline since 2002 for regional tubers, roots, and cereals^30^. Regarding sociobiodiversity foods, availability is low except for yerba mate, representing an average of 7.09g/per capita/day in 2017–2018^31^.

The staple food basket has major implications as it serves as a normative framework for developing policies that favor the production of and access to healthy and sustainable food in Brazil. In the new 2024–2025 Harvest Plan, for example, interest rates for farmers who produce foods included in the new staple food basket, as well as sociobiodiversity, agroecological, and organic products, were reduced^32^. The new staple food basket decree also plays a central role in the ongoing tax reform debate, in which the government proposes that its food products have zero or reduced tax rates, which is even more important when we consider that the low-income population spends almost half of their income on food^33^ and that the relative price of ultra-processed foods has been decreasing substantially since the early 2000s, becoming cheaper than unprocessed or minimally processed foods^34^.

Study limitations include the race/skin color analysis which did not consider households headed by self-identified Yellow and Indigenous heads due to the low representation of these populations in the HSB, especially when the analysis was stratified only for low-income households. Another limitation is that the purchase survey does not consider waste, consumption of food outside the home, and intra-family division of consumption, which can lead to biases in the estimate of what was actually consumed in the households. Moreover, the survey dates back to 2017–2018 and the scenario may have changed, especially in the post-pandemic period. However, the 2017–2018 HSB is the most recent national survey with national representation and a high level of detail, enough to identify the foods studied, in addition to representing most consumption, since eating out represents a smaller part^35^. Additionally, this study can serve as a baseline to identify time trends and assess the potential impacts generated by this 2024 decree based on new household surveys. Notably, this study innovates by focusing on the population usually invisible in research and in including race/skin color as a variable, which is generally underutilized.

In conclusion, our data indicate that in 2017–2018, the foods included in the new staple food basket became the basis of low-income households’ diet.

However, the diet of this population also presented inadequacies, such as high consumption of beef, low consumption of fruits, vegetables and greens and excessive sugar intake. This reinforces the cultural viability of implementing a healthy and sustainable diet aligned with the basket proposal, but the impact of this implementation would be significantly increased if, in addition to access to food, there were combined actions that guaranteed the prioritization of unprocessed or minimally processed foods of plant origin and that supported the population in making healthier choices.

## Data Availability

This study used publicly available secondary data, which do not contain information capable of identifying the participants. https://www.ibge.gov.br/estatisticas/sociais/populacao/24786-pesquisa-de-orcamentosfamiliares-2.html?=&t=microdados

## Footnotes

a Available from: https://doi.org/10.17605/OSF.IO/MU2BR
