# Supplementary material for: Purchase of food from the new staple food basket in low-income Brazilian households
Source: Rev Saude Publica. 2025 Dec 8;59:e43. doi: 10.11606/s1518-8787.2025059006780 (PMC12674687; doi:10.11606/s1518-8787.2025059006780)
Supplement: Translation

# Translation: Aquisição de alimentos da nova cesta básica em domicílios brasileiros de baixa renda

**Authors:** Marcos Anderson Lucas da Silva, Lucas Braga Rodrigues, Samuel Almeida Brito, Luisa Gazola Lage, Maria Laura da Costa Louzada

**Affiliations:** I Universidade de São Paulo. Faculdade de Saúde Pública. Programa de Pós-Graduação em Nutrição em Saúde Pública. São Paulo, SP, Brasil; II Universidade de São Paulo. Núcleo de Pesquisas Epidemiológicas em Nutrição e Saúde. São Paulo, SP, Brasil; III Universidade Federal do Ceará. Instituto de Cultura e Artes. Programa de Pós-Graduação em Gastronomia. Fortaleza, CE, Brasil; IV Universidade de Lisboa. Instituto de Ciências Sociais. Programa de Pós-Graduação em Ciências da Sustentabilidade. Lisboa, Portugal

**Keywords:** Cesta Básica de Alimentos, Alimentação Saudável, Aquisição de Alimentos, Bolsa Família, Baixa Renda

## Abstract

**OBJETIVO:**

Descrever a aquisição dos alimentos que passaram a compor a nova cesta básica de alimentos pelos domicílios em situação de baixa renda no Brasil em 2017–2018.

**MÉTODOS:**

Foram utilizados dados de aquisição domiciliar de alimentos da Pesquisa de Orçamentos Familiares de 2017–2018, realizada pelo Instituto Brasileiro de Geografia e Estatística. Analisaram-se dados de 13.706 domicílios em situação de baixa renda (< ½ salário mínimo *per capita*). Os alimentos foram identificados com base na Portaria do Ministério do Desenvolvimento e Assistência Social, Família e Combate à Fome, que especifica a nova composição da cesta básica de alimentos. As estimativas foram expressas como percentual de participação calórica.

**RESULTADOS:**

Os alimentos que passaram a compor a nova cesta básica representaram 84,1% do total de calorias adquiridas pelos domicílios de baixa renda do Brasil em 2017–2018, sendo essa participação ainda mais expressiva na área rural (88,0%) e nas regiões Norte (88,2%) e Nordeste (85,9%). Os grupos de alimentos com maiores contribuições foram cereais (33,5%), açúcares e óleos/gorduras (21,3%), e carnes e ovos (11,6%). Arroz, milho e outros grãos contribuíram com 19,7%, enquanto os feijões representaram 4,7% das calorias. Carnes de aves e bovina foram os alimentos mais adquiridos do grupo de carnes e ovos, com 5,0% e 4,0% das calorias, respectivamente. Frutas contribuíram com 1,9% e legumes e verduras com 0,7%. Não houve grandes diferenças na aquisição desses alimentos entre as categorias de raça/cor da pele do chefe da família.

**CONCLUSÃO:**

Os dados mostram que, em 2017–2018, os alimentos que passaram a compor a nova cesta eram a base da alimentação dos domicílios em situação de baixa renda, reforçando a viabilidade cultural da sua implementação.

## INTRODUÇÃO

A cesta básica nacional é um conjunto de alimentos essenciais que visa atender às necessidades nutricionais básicas. Instituída em 1938, durante o governo de Getúlio Vargas, a cesta foi concebida como base para o cálculo do salário mínimo, e especificava os tipos e as quantidades necessárias para a subsistência de um trabalhador adulto. Na época, a formação da cesta incluía limitados tipos de carne, cereais, farinhas, leguminosas, hortaliças, legumes e raízes, frutas, além de queijo e manteiga, gorduras, açúcares, café, mate, e leite e ovos^1^.

Com o passar dos anos, a composição dos alimentos da cesta básica foi mudando. Estudo recente, que investigou tributos e normas sanitárias em seis estados do país, identificou políticas públicas que incluíram alimentos ultraprocessados, como embutidos, margarinas, biscoitos e massas prontas para bolo, na cesta básica^2^.

A partir disso, em 2024, foi promulgado o Decreto nº 11.936, de 5 de março do mesmo ano^3^, que delineou novas diretrizes para a composição da cesta básica ao adotar uma concepção mais ampla de alimentação saudável, definindo-a como um “conjunto de alimentos que busca garantir o direito humano à alimentação adequada e saudável, à saúde e ao bem-estar da população brasileira”^3^.

Essa nova perspectiva transcende a mera oferta de calorias suficientes para sanar a fome, considerando também o direito ao acesso regular e permanente a uma dieta que atenda às necessidades biológicas e sociais individuais, respeitando peculiaridades alimentares locais e culturais, bem como as dimensões de gênero, raça e etnia^3,4^. Essas mudanças foram embasadas, majoritariamente, nas recomendações do Guia Alimentar para a População Brasileira, publicado em 2014, que enfatiza o consumo de alimentos *in natura* ou minimamente processados, enquanto desencoraja o consumo de alimentos ultraprocessados^3^. O Guia valoriza, ainda, as tradições culinárias, as diversidades regionais do Brasil e as práticas produtivas sustentáveis^5^.

Acompanhada do novo decreto, foi estabelecida a Portaria nº 966, de 6 de março de 2024^6^, que especifica os alimentos que podem compor a nova cesta básica, abrangendo uma variedade de grupos alimentares, tais como leguminosas, cereais, raízes e tubérculos, legumes e verduras, frutas, castanhas e nozes, carnes e ovos, leites e queijos, além de açúcares, sal, óleos, gorduras, café, chá, mate e especiarias^6^.

É sabido que políticas públicas de segurança alimentar e nutricional tendem a afetar de forma mais intensa a população de mais baixa renda, e um princípio crucial dessas políticas reside na necessidade de priorizar a mitigação de desigualdades sem infringir a autonomia de escolha, ou desrespeitar a cultura alimentar ou hábitos saudáveis, já estabelecidos na população, especialmente em pessoas em situação de maior vulnerabilidade.

Com a promulgação do novo decreto, é essencial compreender até que ponto os alimentos estabelecidos na nova cesta básica já fazem parte da aquisição dos domicílios de baixa renda no Brasil. Diante desse contexto, este estudo tem como objetivo descrever a aquisição dos alimentos que passaram a compor a cesta básica por parte desses domicílios em situação de baixa renda no Brasil, utilizando dados da última edição da Pesquisa de Orçamentos Familiares, realizada em 2017–2018.

## MÉTODOS

### Fonte de Dados

Este estudo utilizou dados do último inquérito de aquisição domiciliar de alimentos da Pesquisa de Orçamentos Familiares (POF) realizada pelo Instituto Brasileiro de Geografia e Estatística (IBGE) no período de julho de 2017 a julho de 2018^7^.

A pesquisa utilizou um plano amostral complexo, agrupado em dois estágios, envolvendo os sorteios dos setores censitários na primeira etapa e dos domicílios na segunda. Os setores censitários são oriundos da amostra mestra do IBGE, agrupados em estratos de domicílios com elevada homogeneidade geográfica e socioeconômica. A construção dos estratos considerou a localização geográfica, a área de residência e, dentro de cada uma, o espectro de variação socioeconômica pela renda do chefe de família. As estimativas obtidas são representativas dos seguintes domínios: Brasil, as cinco macrorregiões (Norte, Nordeste, Centro-Oeste, Sudeste e Sul), área (urbana ou rural), e das 27 Unidades Federativas. A descrição detalhada sobre o processo de amostragem está disponível na publicação do IBGE^7^.

As informações neste estudo referem-se às aquisições domiciliares de alimentos durante sete dias consecutivos, registradas pelos moradores do domicílio ou por entrevistador do IBGE, considerando aquisições monetárias (dinheiro, cartões de crédito e débito) e não monetárias (doação, troca, produção própria). A coleta foi distribuída nos quatro trimestres do ano, incorporando a variedade sazonal, à qual as despesas estão sujeitas. Os agregados de domicílios gerados no plano amostral (estratos) foram usados como unidade de estudo. De 2017 a 2018, os 57.920 domicílios estudados resultaram em 575 estratos com média de 86,5 domicílios (variando de 16 a 524). Adicionalmente, foram utilizadas informações socioeconômicas e demográficas, coletadas pela POF por meio de questionários padronizados^7^.

### Organização dos Dados e Criação das Variáveis de Estudo

#### Alimentos da nova cesta básica

Os alimentos que passaram a compor a nova cesta básica foram identificados na última POF com base na Portaria do Ministério do Desenvolvimento e Assistência Social, Família e Combate à Fome (MDS) nº 966, de 6 de março de 2024, que define uma relação de alimentos que podem compor a nova cesta básica^6^.

A nova composição inclui alimentos *in natura* ou minimamente processados (aqueles obtidos diretamente de plantas, de animais ou de fungos e adquiridos para consumo sem que tenham sofrido qualquer alteração após deixarem a natureza ou que tenham sido submetidos a processamentos que não adicionam sal, açúcares, óleos ou gorduras), ingredientes culinários (produtos extraídos de alimentos *in natura*, como óleos, gorduras e açúcares, ou da natureza, como o sal) e alguns alimentos processados (aqueles fabricados com a adição de sal, açúcar, óleos ou gorduras a alimentos *in natura* ou minimamente processados, como sardinha e atum enlatado, queijos)^6^. Está vedada a inclusão de alimentos ultraprocessados, definidos como formulações industriais feitas tipicamente com muitos ingredientes e com diversas etapas e tipos de processamentos, com pouca ou nenhuma presença de alimentos *in natura* e caracterizadas pela presença de aditivos alimentares cosméticos ou substâncias de uso industrial^6^. Para este estudo, foram considerados os dez grupos de alimentos destacados na Portaria do MDS nº 966, de 6 de março de 2024^6^: 1) leguminosas; 2) cereais; 3) raízes e tubérculos; 4) legumes e verduras; 5) frutas; 6) carnes e ovos; 7) leites e queijos; 8) ingredientes culinários (identificado na Portaria como “Açúcares, sal, óleos e gorduras”); 9) castanhas e nozes; e 10) café, chá, mate e especiarias. Para uma análise mais detalhada, criaram-se subgrupos dos alimentos, com exceção do grupo de castanhas e nozes. No Quadro é possível ver uma lista extensa de exemplos de alimentos de cada grupo.

**Quadro:** Gruposa e subgrupos dos alimentos da nova cesta básica.

Grupos	Subgrupos	Exemplos de alimentos
Cereais	Arroz, milho e outros grãos	Arroz branco, integral ou parabolizado, a granel ou embalado; milho em grão ou na espiga, grãos de trigo, aveia
	Pães	Pães feitos somente de farinha de trigo e/ou outras farinhas feitas de alimentos *in natura* e minimamente processadas, leveduras, água, sal
	Farinhas de milho, de trigo e outros cereais	Farinhas de milho, de trigo, arroz, aveia, linhaça, sorgo
	Macarrão	Macarrão ou massas frescas ou secas feitas com essas farinhas/sêmola, água e/ou ovos e/ou outros alimentos *in natura* ou minimamente processado
Ingredientes culinários	Óleos vegetais	Óleos de soja, de girassol, de milho, de dendê, dentre óleos vegetais; azeite de oliva
	Açúcares	Açúcar de mesa branco, demerara ou mascavo e mel
	Manteiga	Com ou sem sal
	Banha	Banha de porco
Carnes e ovos	Carne de aves	Frango e outros aves
	Carne bovina	Carne de boi
	Ovos	Galinha e outras aves
	Carne suína	Carne de porco
	Pescado	Todos os peixes de consumo habitual
	Sardinha e atum enlatado	Peixe sardinha ou atum enlatado
	Carne ovina e caprina	Cordeiro, carneiro, cabrito
	Outras carnes *in natura* ou minimamente processadas	Vísceras e outras partes e carnes pouco consumidas
Leite e queijos	Leite	Leite fluido pasteurizado ou industrializado, na forma de ultrapasteurizado, leite em pó, integral, semidesnatado ou desnatado
	Queijo	Queijos feitos de leite e sal e microrganismos usados para fermentar o leite
	Iogurte natural	Iogurte natural sem adição de açúcar, edulcorante e/ou aditivos que modifiquem as características sensoriais do produto
Leguminosas	Feijões	Feijões de todas as cores (preto, branco, roxo, mulatinho, verde, carioca, fradinho, rajado, manteiga, jalo, de-corda, andú, dentre outros)
	Outras leguminosas	Ervilha, lentilha, grão-de-bico, fava, guandu, orelha-de-padre
Raízes e tubérculos	Farinha de mandioca e de outras raízes e tubérculos	Farinhas minimamente processadas de mandioca, dentre outras farinhas
	Mandioca, batata e outras raízes e tubérculos	Ariá, batata-inglesa, batata-doce, batata-baroa/mandioquinha, batata-crem, cará, cará-amazônico, cará-de-espinho, inhame, mandioca/macaxeira/aipim, e outras raízes e tubérculos *in natura* ou embalados, fracionados, refrigerados ou congelados
	Preparações derivadas da mandioca	Farinha de carimã, farinha de uarini; maniçoba e tucupi, farinha/gomo de tapioca, dentre outros
Frutas	*In natura* (frescas ou secas)	Frutas *in natura* ou frutas frescas ou secas embaladas, fracionadas, refrigeradas ou congelada Exemplos: abacate, abacaxi, abiu, abricó, açaí, açaí-solteiro, acerola, ameixa, amora, araçá, araçá-boi, araçá-pera, araticum, aroeira-pimenteira, arumbeva, atemoia, babaçu, bacaba, bacupari, bacuri, banana, baru, biribá, brejaúva, buriti, butiá, cacau, cagaita, cajarana, cajá, caju, caju do cerrado, cajuí, cambuci, cambuí, camu-camu, caqui, carambola, cereja-do-rio-grande, ciriguela, coco, coco-cabeçudo, coco-indaiá, coquinho-azedo, coroa-de-frade, croá, cubiu, cupuaçu, cupuí, cutite, curriola, figo, fisalis, fruta-pão, goiaba, goiaba-serrana, graviola, guabiroba, grumixama, guapeva, guaraná, inajá, ingá, jaca, jabuticaba, jambo, jambolão, jaracatiá, jatobá, jenipapo, juá, juçara, jurubeba, kiwi, laranja, limão, lobeira, maçã, macaúba, mama-cadela, mamão, mandacaru, manga, mangaba, mapati, maracujá, marmelada-de-cachorro, melancia, melão, mexerica/tangerina/ bergamota, morango, murici, nectarina, pajurá, patauá, pequi, pera, pera-do-cerrado, pêssego, piquiá, pinha/fruta do conde, pinhão, pitanga, pitomba, pupunha, romã, sapucaia, sapoti, sapota, seriguela, sete-capotes, sorva, tamarindo, taperebá, tucumã, umari, umbu, umbu-cajá, uva, uvaia, uxi, xixá, dentre outros
	Polpa	Polpas de frutas sem adição de açúcar
Legumes e verduras	*In natura*	Legumes e verduras *in natura* ou embalado, fracionados, refrigerados ou congelados, tais como abóbora/jerimum, abobrinha, acelga, agrião, alface, almeirão, alho, alho-poró, azedinha, berinjela, beterraba, beldroega, bertalha, brócolis, broto-de-bambu, capicoba, capuchinha, carrapicho-agulha, caruru, catalonha, cebola, cebolinha, cenoura, cheiro-verde, chicória, chicória-paraense/chicória-do-pará, chuchu, couve, couve-flor, croá, crem, dente-de-leão, escarola, espinafre, gueroba, gila, guariroba, jambu, jiló, jurubeba, major-gomes, maxixe, mini-pepininho, mostarda, muricato, ora-pro-nóbis, palma, pepino, peperômia, pimentão, puxuri, quiabo, radite, repolho; rúcula, salsa, serralha, taioba, tomate, urtiga, vinagreira, vagem, dentre outros
	Conservas e extratos	Cenoura, pepino, palmito, cebola, couve-flor, dentre outros legumes e verduras, preservados em salmoura ou em solução de sal e vinagre; extrato ou concentrados de tomate e/ou outros alimentos *in natura* e minimamente processado (com sal e ou açúcar)^a^
Café, chá, mate e especiarias	Especiarias	Pimenta, pimenta-do-reino, canela, cominho, cravo-da-índia, coentro, noz-moscada, gengibre, açafrão, cúrcuma, dentre outros
	Café	Café moído, em grão
	Mate	Erva-mate *in natura*
	Chá	Camomila, erva-doce, canela, entre outros chás
Castanhas e nozes	-	Amendoim, castanha-de-caju, castanha de baru, castanha-do-brasil (castanha-do-pará), castanha-de-cutia, castanha-de-galinha, chichá, licuri, macaúba, e outras oleaginosas sem sal ou açúcar

Fonte: elaborado a partir da Portaria nº 966/2024.

^a^ Portaria do Ministério do Desenvolvimento e Assistência Social, Família e Combate à Fome nº 966, de 6 de março de 2024.

Fatores de correção foram aplicados para excluir a fração não comestível dos alimentos^8^. Em seguida, as quantidades dos alimentos adquiridos (quilogramas ou litros) foram convertidas em calorias, empregando-se a Tabela Brasileira de Composição de Alimentos da Universidade de São Paulo^9^.

## Variáveis Socioeconômicas e Demográficas

As variáveis socioeconômicas e demográficas utilizadas neste estudo foram: sexo (masculino e feminino), raça/cor autodeclarada pelo chefe da família (branco, pardo, preto, amarelo, indígena), escolaridade do chefe da família (sem instrução, fundamental incompleto, fundamental completo, médio incompleto, médio completo, superior incompleto ou mais)*,* presença de crianças de até 5 anos no domicílio (sim ou não), recebe Bolsa Família (sim ou não), área de residência (urbana e rural), e Grandes Regiões (Norte, Nordeste, Centro-Oeste, Sudeste e Sul), além da renda média mensal familiar *per capita*.

A renda mensal familiar *per capita* é uma estimativa que corresponde ao somatório dos rendimentos totais brutos monetários dos componentes das unidades de consumo em um mês dividido pelo número de moradores do domicílio. Os rendimentos totais foram criados a partir das informações de rendimento monetário e não monetário, incluindo o recebimento de Bolsa Família ou outros programas de transferência de renda.

## Análise de Dados

Foram incluídos no estudo apenas os domicílios da POF em situação de baixa renda, definidos como aqueles com renda familiar mensal *per capita* de até meio salário mínimo^10^, utilizando como referência o salário mínimo mensal de 2018, equivalente a 954 reais (R$)^11^, correspondendo a R$ 477 para meio salário. Ao todo, analisaram-se 13.706 domicílios (23,66% da amostra total da POF).

Primeiramente, foram descritas as características socioeconômicas e demográficas dos domicílios, apresentando as proporções (%) com seus respectivos intervalos de confiança de 95% (IC95%).

Em seguida, estimou-se a participação da aquisição dos grupos e subgrupos de alimentos da nova cesta básica. As estimativas foram descritas a partir do percentual médio de calorias totais (% kcal), com seus respectivos intervalos de confiança de 95%, considerando o Brasil como um todo e segundo a área dos domicílios (urbano e rural), as grandes regiões do país e a raça/cor autodeclarada pelo chefe da família.

Nas análises por raça/cor da pele, foram avaliados somente os domicílios chefiados por pessoas pretas, pardas e brancas, e as categorias amarela e indígena não foram incluídas devido ao baixo tamanho amostral (menos de 1% do total dos domicílios). Quanto aos domicílios chefiados por pessoas pretas e pardas, eles foram avaliados separadamente e em uma categoria conjunta de raça/cor da pele negra.

Por fim, aplicaram-se fatores de ponderação considerando a estrutura amostral, permitindo a extrapolação dos resultados para toda a população brasileira. As análises foram realizadas no *software* Stata 18.0/SE e os resultados foram revisados por todos os autores.

## RESULTADOS

No Brasil, em 2017–2018, a maioria dos chefes dos domicílios em situação de baixa renda era do sexo masculino (59,0%), que se autodeclaravam da raça/cor da pele parda (54,4%) e tinham ensino fundamental incompleto (36,0%). A maior parte dos domicílios recebiam Bolsa Família (60,2%) e mais de um terço (36,4%) tinha crianças de até cinco anos. Os domicílios estavam localizados predominantemente na área urbana (73,2%) e na região Nordeste (47,4%) (Tabela 1).

**Tabela 1:** Características socioeconômicas dos domicílios em situação de baixa renda. Brasil, 2017–2018.

Características socioeconômicas	%	IC95%
Sexo do chefe da família		
Masculino	59,0	55,9–64,0
Feminino	41,0	36,1–44,1
Raça/cor autodeclarada pelo chefe da família		
Parda	54,4	48,4–60,2
Branca	34,3	28,8–40,2
Preta	10,4	7,4–14,5
Amarela	0,5	0,0–1,0
Indígena	0,4	0,1–0,9
Escolaridade do chefe da família		
Sem instrução	12,9	9,8–16,1
Fundamental incompleto	36,0	30,4–41,5
Fundamental completo	8,7	6,1–11,3
Médio incompleto	5,9	4,2–7,6
Médio completo	23,0	21,3–24,7
Superior incompleto ou mais	11,6	9,5–13,7
Presença de crianças de até 5 anos		
Sim	36,4	30,5–42,3
Não	63,6	57,7–69,5
Bolsa Família		
Sim	60,2	54,3–65,9
Não	39,8	34,1–45,7
Área de moradia		
Urbano	73,2	67,8–78,0
Rural	26,8	22,0–32,2
Região do País		
Nordeste	47,4	41,5–53,3
Sudeste	26,3	21,2–32,1
Norte	13,8	9,8–19,1
Sul	7,1	5,2–9,6
Centro-Oeste	5,3	3,9–7,5

IC95%: Intervalo de confiança de 95%.

A Tabela 2 descreve a participação média (%) de calorias dos grupos e subgrupos de alimentos que passaram a compor a nova cesta básica em domicílios de baixa renda para o país como um todo e segundo a área dos domicílios. Os alimentos que compõem a nova cesta básica representaram 84,1% do total de calorias adquiridas pelos domicílios em situação de baixa renda no Brasil, sendo superior na área rural (88,1%) em relação à área urbana (82,7%). Os grupos de alimentos que apresentaram as maiores contribuições no país como um todo foram: cereais (33,5%), ingredientes culinários (21,3%) e carne e ovos (11,6%), seguidos de leites e queijos (5,1%) e leguminosas (5,0%). No grupo de cereais, o subgrupo de arroz, milho e outros grãos contribuíram com 19,7%, enquanto o feijão, do grupo de leguminosas, representou 4,7% das calorias. Em relação ao grupo de ingredientes culinários, óleos vegetais e açúcares contribuíram com cerca de 10,3% e 10,6%, respectivamente. No grupo de carnes e ovos, as carnes mais consumidas foram as de aves e bovina, com 5,0% e 4,0% das calorias, respectivamente. Frutas representaram 1,9%, enquanto legumes e verduras 0,8% das calorias.

**Tabela 2:** Participação média (%) de calorias dos grupos e subgrupos de alimentos que compõem a nova cesta básica em domicílios de baixa renda para o país como um todo e segundo área de residência. Brasil, 2017–2018.

Grupos e subgrupos dos alimentos da nova cesta básica	Participação média (%) de calorias					
	Brasil		Urbano		Rural	
	%	IC95%	%	IC95%	%	IC95%
**Cereais**	**33,5**	**32,4–34,6**	**33,3**	**32,1–34,5**	**34,0**	**31,0–36,6**
Arroz, milho e outros grãos	19,7	18,4–21,0	18,5	17,1–20,0	22,7	20,1–25,4
Pães^a^	8,2	7,5–8,8	9,6	8,9–10,32	4,3	3,7–4,9
Farinhas de milho, de trigo e outros cereais	3,0	2,7–3,3	2,5	2,2–2,8	4,4	3,5–5,4
Macarrão^b^	2,7	2,5–2,9	2,7	2,5–3,0	2,6	2,2–3,0
**Ingredientes culinários**	**21,3**	**20,5–22,1**	**20,6**	**19,6–21,5**	**23,3**	**22,2–24,5**
Óleos vegetais	10,3	9,8–10,9	10,1	9,4–10,9	10,9	10,1–11,7
Açúcares	10,6	10,0–11,1	10,0	9,3–10,7	12,1	11,3–12,9
Manteiga	0,3	0,2–0,4	0,3	0,2–0,4	0,2	0,1–0,3
Banha	0,1	0,0–0,2	0,1	0,0–0,2	0,1	0,0–0,3
**Carnes e ovos**	**11,6**	**11,0–12,1**	**11,3**	**10,7–11,9**	**12,3**	**11,5–13,2**
Carne de aves	5,0	4,7–5,3	4,8	4,4–5,2	5,4	4,9–5,9
Carne bovina	4,0	3,8–4,3	4,1	3,8–4,4	3,9	3,4–4,3
Ovos	0,9	0,7–0,9	0,9	0,8–1,0	0,9	0,7–1,1
Carne suína	0,8	0,8–1,0	0,8	0,6–0,9	0,8	0,6–1,0
Pescado	0,7	0,5–0,8	0,5	0,4–0,6	1,1	0,7–1,4
Sardinha e atum enlatado	0,1	0,1–0,1	0,1	0,1–0,1	0,1	0,1–0,1
Carne ovina e caprina	0,1	0,1–0,1	0,1	0,0–0,1	0,2	0,1–0,3
Outras carnes *in natura* ou minimamente processadas	0,0	0,0–0,1	0,0	0,0–0,1	0,0	0,0–0,1
**Leite e queijos**	**5,1**	**4,8–5,4**	**5,4**	**5,0–5,8**	**4,4**	**4,7–5,0**
Leite	4,6	4,3–4,9	4,7	4,4–5,1	4,1	3,5–4,7
Queijo^c^	0,5	0,4–0,6	0,6	0,5–0,7	0,3	0,2–0,3
Iogurte natural^d^	0,0	0,0–0–0	0,0	0,0–0,0	0,0	0,0–0,0
**Leguminosas**	**5,0**	**4,7–5,3**	**4,9**	**4,6–5,3**	**5,3**	**4,6–5,9**
Feijões	4,7	4,4–5,0	4,6	4,3–4,9	4,9	4,3–5,5
Outras leguminosas	0,4	0,3–0,5	0,4	0,2–0,5	0,4	0,2–0,6
**Raízes e tubérculos**	**4,8**	**4,0–5,5**	**4,2**	**3,6–4,8**	**6,4**	**4,3–8,4**
Farinha de mandioca e de outras raízes e tubérculos	3,2	2,5–4,0	2,7	2,1–3,3	4,8	2,6–6,9
Mandioca, batata e outras raízes e tubérculos	0,9	0,8–1,0	0,9	0,8–1,0	0,8	0,7–1,0
Preparações derivadas da mandioca^e^	0,6	0,5–0,7	0,6	0,5–0,7	0,7	0,5–1,0
**Frutas**	**1,9**	**1,8–2,1**	**2,0**	**1,8–2,2**	**1,6**	**1,3–1,9**
*In natura* (frescas ou secas)	1,9	1,7–2,1	2,0	1,8–2,2	1,6	1,3–1,9
Polpa^f^	0,0	0,0–0,0	0,0	0,0–0,0	0,0	0,0–0,1
**Legumes e verduras**	**0,8**	**0,7–0,8**	**0,8**	**0,7–0,9**	**0,6**	**0,5–0,7**
*In natura*	0,7	0,7–0,8	0,8	0,7–0,9	0,6	0,5–0,7
Conservas e extratos	0,0	0,0–0,0	0,0	0,0–0,0	0,0	0,0–0,0
**Café, chá, mate e especiarias**	**0,2**	**0,1–0,2**	**0,2**	**0,1–0,2**	**0,2**	**0,1–0,2**
Especiarias^g^	0,1	0,1–0,1	0,1	0,1–0–1	0,1	0,1–0,2
Café	0,1	0,0–0,1	0,1	0,1–0,1	0,1	0,1–0,1
Mate	0,0	0,0–0,0	0,0	0,0–0,0	0,0	0,0–0,0
Chá	0,0	0,0–0,0	0,0	0,0–0,0	0,0	0,0–00
**Castanhas e nozes** ^h^	**0,0**	**0,0–0,0**	**0,0**	**0,0–0,0**	**0,0**	**0,0–0,0**
Total	84,1	83,4–84,9	82,7	81,8–83,6	88,1	87,2–88,9

IC95%: intervalo de confiança de 95%.

^a^ Feitos somente de farinha de trigo e/ou outras farinhas feitas de alimentos *in natura* e minimamente processados, leveduras, água, sal e/ou outros alimentos *in natura* e minimamente processados.

^b^ Massas frescas ou secas feitas com farinhas/sêmola, água e/ou ovos e/ou outros alimentos *in natura* ou minimamente processados.

^c^ Feitos de leite e sal e microrganismos usados para fermentar o leite.

^d^ Sem adição de açúcar, edulcorante e/ou aditivos que modifiquem as características sensoriais do produto.

^e^ Maniçoba, tucupi e farinha/gomo de tapioca, entre outras.

^f^ Sem adição de açúcar.

^g^ Pimenta, noz-moscada, açafrão, entre outros.

^h^ Sem sal ou açúcar.

Domicílios da área rural apresentaram maiores participações calóricas, em relação à área urbana, de arroz, milho e outros grãos (22,7% *versus* 18,5%), farinhas de milho, de trigo e outros cereais (4,4% *versus* 2,5%), açúcares (12,1% *versus* 10,0%), pescados (1,1% *versus* 0,5%) e farinha de mandioca ou de outras raízes e tubérculos (4,8% *versus* 2,7%). Por outro lado, domicílios da área urbana apresentaram, em relação à área rural, maior participação de pães (9,6% *versus* 4,3%), leite (4,7% *versus* 4,1%), queijos (0,6% *versus* 0,3%), frutas (2,0% *versus* 1,6%) e legumes e verduras (0,8% *versus* 0,6%).

A Tabela 3 mostra a participação média (%) de calorias dos grupos e subgrupos de alimentos que passaram a compor a nova cesta básica em domicílios de baixa renda de acordo com as grandes regiões do país. A participação dos alimentos foi maior na região Norte (88,2%) e Nordeste (85,9%), intermediária no Centro-Oeste (82,8%) e menor no Sudeste (80,4%) e Sul (79,2%). A região Norte se destacou, em relação às outras regiões, pela elevada contribuição de farinha de mandioca e de outras raízes e tubérculos (10,2%), carne de aves (6,8%) e pescado (2,1%). O Sul se destacou pela maior participação calórica de farinhas de milho, trigo e outros cereais (7,3%), o Sudeste pelo de leite (6,1%) e o Centro-Oeste pelo arroz, milho e outros grãos (24,3%). A maior participação calórica de feijões foi no Nordeste (5,4%) e a menor foi no Sul (3,0%). A participação calórica de frutas variou de 2,3% no Norte a 1,7% no Nordeste, enquanto a participação de legumes e verduras variou de 0,9% no Centro-Oeste a 0,4% no Norte.

**Tabela 3:** Participação média (%) de calorias dos grupos e subgrupos de alimentos da nova cesta básica em domicílios de baixa renda de acordo com as grandes regiões do país. Brasil, 2017–2018.

Grupos e subgrupos dos alimentos da nova cesta básica	Participação média (%) de calorias									
	Norte		Nordeste		Sudeste		Sul		Centro-Oeste	
	%	IC95%	%	IC95%	%	IC95%	%	IC95%	%	IC95%
**Cereais**	**29,4**	**25,4–33,4**	**35,8**	**34,3–37,2**	**32,2**	**30,2–34,1**	**30,1**	**27,9–32,3**	**34,8**	**30,9–38,8**
Arroz, milho e outros grãos	18,9	14,9–22,9	20,7	18,7–22,8	18,5	16,6–20,3	15,2	13,1–17,3	24,3	20,6–27,9
Pães^a^	6,8	4,8–8,8	8,6	7,8–9,4	8,9	7,5–10,3	5,2	4,0–6,5	7,5	3,9–11,0
Farinhas de milho, de trigo e outros cereais	1,8	1,4–2,3	3,4	2,8–4,0	2,0	1,6–2,4	7,3	5,6–9,0	1,7	1,0–2,4
Macarrão^b^	1,8	1,6–2,1	3,0	2,7–3,4	2,8	2,3–3,2	2,4	1,9–2,9	1,4	1,2–1,7
**Ingredientes culinários**	**20,7**	**19,1–22,3**	**19,9**	**19,1–20,8**	**23,3**	**21,3–25,3**	**23,8**	**21,0–26,6**	**21,8**	**18,7–24,9**
Óleos vegetais	10,6	9,1–12,0	8,8	8,1–9,3	12,1	10,7–13,5	12,9	10,4–15,3	11,9	10,1–13,7
Açúcares	9,8	8,8–10,8	10,9	10,3–11,42	10,8	9,2–12,5	10,3	8,5–12,0	9,6	7,5–11,7
Manteiga	0,3	0,2–0,5	0,3	0,3–0,4	0,2	0,1–0,4	0,1	0,0–0,1	0,2	0,0–0,3
Banha	0,0	0,0–0,0	0,0	0,0–0,0	0,2	0,0–0,4	0,6	0,2–1,0	0,1	0,0–0,2
**Carnes e ovos**	**16,0**	**14,8–17,1**	**12,1**	**11,6–12,7**	**8,3**	**7,5–9,1**	**11,1**	**9,5–12,6**	**11,9**	**10,4–13,4**
Carne de aves	6,8	6,1–7,6	5,7	5,4–6,1	3,0	2,6–3,5	4,1	3,3–4,9	3,8	3,1–4,5
Carne bovina	5,4	4,7–6,1	3,9	3,6–4,2	3,0	2,6–3,5	4,6	3,6–5,5	5,9	4,8–7,0
Ovos	0,6	0,5–0,7	1,0	0,9–1,1	0,9	0,7–1,0	0,8	0,6–1,0	0,7	0,6–0,8
Carne suína	0,3	0,2–0,5	0,6	0,4–0,7	1,1	0,9–1,4	1,5	1,0–2,0	1,2	0,5–2,0
Pescado	2,2	1,6–2,7	0,6	0,5–0,8	0,2	0,1–0,3	0,0	0,0–0,1	0,1	0,0–0,3
Sardinha e atum enlatado	0,1	0,0–0,1	0,1	0,1–0,1	0,0	0,0–0,1	0,0	0,0–0,1	0,1	0,0–0,1
Carne ovina e caprina	0,2	0,1–0,3	0,1	0,1–0,2	0,0	0,0–0,0	0,0	0,0–0,1	0,1	0,0–0,2
Outras carnes *in natura* ou minimamente processadas	0,3	0,1–0,6	0,0	0,0–0,0	-	-	-	-	-	-
**Leite e queijos**	**3,9**	**3,2–4,5**	**4,4**	**4,1–4,7**	**6,8**	**6,0–7,6**	**6,2**	**5,4–7,0**	**4,5**	**3,7–5,3**
Leite	3,6	3,0–4,2	3,9	3,6–4,2	6,1	5,3–7,0	5,4	4,7–6,2	4,0	3,2–4,8
Queijo^c^	0,2	0,1–0,3	0,5	0,4–0,6	0,7	0,5–0,8	0,7	0,5–0,9	0,5	0,3–0,6
Iogurte natural^d^	0,0	0,0–0,0	0,0	0,0–0,0	0,0	0,0–0,0	0,0	0,0–0,0	0,0	0,0–0,0
**Leguminosas**	**3,6**	**3,3–4,0**	**5,9**	**5,5–6,3**	**4,9**	**4,2–5,6**	**3,6**	**2,9–4,3**	**4,1**	**3,2–4,9**
Feijões	3,4	3,0–3,7	5,4	4,9–5,9	4,6	5,0–5,8	3,0	2,3–3,7	3,8	2,9–4,6
Outras leguminosas	0,3	0,2–0,4	0,4	0,3–0,6	0,3	0,1–0,4	0,5	0,0–1,0	0,3	0,1–0,5
**Raízes e tubérculos**	**11,6**	**8,5–14,7**	**5,1**	**4,6–5,6**	**2,0**	**1,6–2,3**	**1,7**	**1,3–2,0**	**2,4**	**1,6–3,1**
Farinha de mandioca e de outras raízes e tubérculos	10,2	6,9–13,6	3,4	2,9–3,9	0,7	0,5–0,9	0,2	0,1–0,4	0,6	0,3–0,8
Mandioca, batata e outras raízes e tubérculos	0,5	0,3–0,6	0,8	0,7–1,0	1,0	0,9–1,2	1,3	1,0–1,5	1,3	0,8–1,8
Preparações derivadas da mandioca^e^	0,9	0,6–1,2	0,9	0,7–1,1	0,2	0,1–0,3	0,2	0,1–0,3	0,5	0,1–0,9
**Frutas**	**2,4**	**1,7–3,0**	**1,8**	**1,6–1,9**	**1,9**	**1,6–2,3**	**1,8**	**1,4–2,1**	**2,3**	**1,7–2,9**
*In natura* (frescas ou secas)	2,3	1,6–3,0	1,7	1,6–1,9	1,9	1,6–2,3	1,8	1,4–2,1	2,3	1,7–3,0
Polpa^f^	0,1	0,0–0,1	0,0	0,0–0,0	0,0	0,0–0,0	-	-	0,0	0,0–0,0
**Legumes e verduras**	**0,4**	**0,4–0,5**	**0,8**	**0,7–0,8**	**0,9**	**0,8–1,0**	**0,8**	**0,7–1,0**	**1,0**	**0,7–1,2**
*In natura*	0,4	0,4–0,5	0,7	0,7,0,8	0,9	0,7–1,0	0,8	0,6–0,9	0,9	0,7–1,2
Conservas e extratos	0,0	0,0–0,0	0,0	0,0–0,0	0,0	0,0–0,0	0,0	0,0–0,1	0,0	0,0–0,0
**Café, chá, mate e especiarias**	**0,2**	**0,1–0,2**	**0,2**	**0,2–0,2**	**0,1**	**0,1–0,2**	**0,1**	**0,1–0,2**	**0,1**	**0,1–0,2**
Especiarias^g^	0,1	0,1–0,2	0,1	0,1–0,2	0,1	0,0–0,1	0,1	0,0–0,1	0,1	0,1–0,1
Café	0,1	0,0–0,1	0,1	0,1–0,2	0,1	0,0–0,1	0,1	0,0–0,1	0,1	0,0–0,1
Mate	-	-	-	-	0,0	0,0–0,0	0,0	0,0–0,0	0,0	0,0–0,1
Chá	0,0	0,0–0,0	0,0	0,0–0,0	0,0	0,0–0,0	0,0	0,0–0,0	0,0	0,0–0,0
**Castanhas e nozes** ^h^	**0,0**	**0,0–0,1**	**0,0**	**0,0–0,0**	**0,0**	**0,0–0,0**	**0,0**	**0,0–0,1**	**0,0**	**0,0–0,1**
Total	88,2	87,4–89,1	85,9	85,0–86,8	80,4	78,7–82,1	79,2	77,1–81,2	82,8	80,4–85,3

IC95%: intervalo de confiança de 95%.

^a^ Feitos somente de farinha de trigo e/ou outras farinhas feitas de alimentos *in natura* e minimamente processados, leveduras, água, sal e/ou outros alimentos *in natura* e minimamente processados.

^b^ Massas frescas ou secas feitas com farinhas/sêmola, água e/ou ovos e/ou outros alimentos *in natura* ou minimamente processados.

^c^ Feitos de leite e sal e microrganismos usados para fermentar o leite.

^d^ Sem adição de açúcar, edulcorante e/ou aditivos que modifiquem as características sensoriais do produto.

^e^ Maniçoba, tucupi e farinha/gomo de tapioca, entre outras.

^f^ Sem adição de açúcar.

^g^ Pimenta, noz-moscada, açafrão, entre outros.

^h^ Sem sal ou açúcar.

A Tabela 4 apresenta a participação média (%) de calorias dos grupos e subgrupos de alimentos que passaram a compor a nova cesta básica em domicílios de baixa renda de acordo com a raça/cor da pele autodeclarada pelo chefe da família. Houve pouca diferença na participação de alimentos entre as categorias de raça/cor da pele do chefe da família, variando entre 85,2% das calorias entre pardos a 82,5% entre brancos. A participação calórica de carnes e ovos foi levemente superior nos domicílios chefiados por pessoas negras (12,1%,) em relação ao observado nos domicílios chefiados por pessoas brancas (10,5%).

**Tabela 4:** Participação média (%) de calorias dos grupos e subgrupos de alimentos da nova cesta básica em domicílios de baixa renda de acordo com a raça/cor da pele autodeclarada* do chefe da família. Brasil, 2017–2018.

Grupos e subgrupos dos alimentos da nova cesta básica	Participação média (%) de calorias							
	Branca		Negra**		Preta		Parda	
	%	IC95%	%	IC95%	%	IC95%	%	IC95%
**Cereais**	**32,6**	**30,5–34,7**	**34,0**	**32,7–35,5**	**32,1**	**29,6–34,6**	**34,4**	**32,9–35,9**
Arroz, milho e outros grãos	18,8	16,8–20,8	20,2	18,6–21,8	18,7	16,5–20,9	20,5	18,6–22,3
Pães^a^	7,7	6,8–8,7	8,3	7,5–9,2	7,7	6,0–9,3	8,5	7,5–9,4
Farinhas de milho, de trigo e outros cereais	3,4	2,7–4,0	2,8	2,4–3,2	2,6	1,5–3,7	2,9	2,4–3,3
Macarrão^b^	2,7	2,4–3,0	2,7	2,4–2,9	3,1	2,4–3,8	2,6	2,3–2,8
**Ingredientes culinários**	**22,0**	**20,5–23,5**	**21,0**	**20,1–21,8**	**20,6**	**18,7–22,4**	**21,0**	**20,1–22,0**
Óleos vegetais	10,9	9,9–11,9	10,0	9,4–10,7	10,0	8,3–11,6	10,1	9,3–10,8
Açúcares	10,7	9,6–11,8	10,5	9,9–11,1	10,1	8,6–11,5	10,6	9,9–11,3
Manteiga	0,2	0,1–0,3	0,3	0,2–0,4	0,4	0,1–0,6	0,3	0,2–0,4
Banha	0,2	0,0–0,4	0,1	0,0–0,1	0,2	0,1–0,3	0,0	0,0–0,0
**Carnes e ovos**	**10,5**	**9,7–11,3**	**12,1**	**11,4–12,8**	**11,5**	**10,0–12,9**	**12,2**	**11,5–13,0**
Carne de aves	4,4	3,8–4,9	5,3	4,9–5,6	4,9	4,1–5,7	5,4	5,0–5,8
Carne bovina	3,7	3,3–4,1	4,2	3,9–4,5	4,4	3,7–5,1	4,2	3,8–4,5
Ovos	0,9	0,8–1,0	0,7	0,8–1,0	1,0	0,7–1,3	0,9	0,6–1,2
Carne suína	0,9	0,7–1,2	0,9	0,6–0,8	0,5	0,2–0,7	0,7	0,6–0,8
Pescado	0,4	0,2–0,6	0,8	0,6–1,0	0,5	0,3–0,7	0,9	0,7–1,1
Sardinha e atum enlatado	0,1	0,0–0,1	0,1	0,0–0,1	0,1	0,0–0,1	0,1	0,0–0,1
Carne ovina e caprina	0,1	0,0–0,1	0,1	0,0–0,1	0,1	0,0–0,2	0,1	0,0–0,1
Outras carnes *in natura* ou minimamente processadas	0,0	0,0–0,0	0,1	0,0–0,1	0,1	0,0–0,2	0,1	0,0–0,1
**Leite e queijos**	**5,6**	**4,9–6,2**	**4,8**	**4,4–5,2**	**5,8**	**4,3–7,3**	**4,6**	**4,2–5,0**
Leite	5,9	4,3–5,5	4,3	4,0–4,7	5,3	3,9–6,6	4,2	3,8–4,5
Queijo^c^	0,7	0,5–0,8	0,5	0,4–0,5	0,5	0,3–0,7	0,4	0,4–0,5
Iogurte natural^d^	0,0	0,0–0,0	0,0	0,0–0,0	0,0	0,0–0,0	0,0	0,0–0,0
**Leguminosas**	**4,8**	**4,3–5,3**	**5,2**	**4,8–5,5**	**5,7**	**4,8–6,7**	**5,1**	**4,7–5,4**
Feijões	4,5	4,0–5,0	4,8	4,4–5,1	5,4	4,5–6,3	4,6	4,3–5,0
Outras leguminosas	0,3	0,2–0,5	0,4	0,3–0,5	0,3	0,1–0,4	0,4	0,3–0,5
**Raízes e tubérculos**	**4,2**	**2,4–5,9**	**5,1**	**4,4–5,8**	**5,1**	**3,5–6,8**	**5,1**	**4,3–5,8**
Farinha de mandioca e de outras raízes e tubérculos	2,7	0,8–4,5	3,5	2,8–4,2	3,7	1,9–5,4	3,5	2,8–4,2
Mandioca, batata e outras raízes e tubérculos	1,0	0,9–1,2	0,8	0,7–0,9	0,9	0,6–1,1	0,8	0,7–0,9
Preparações derivadas da mandioca^e^	0,5	0,3–0,6	0,7	0,6–0,9	0,6	0,2–0,9	0,8	0,8–2,2
**Frutas**	**1,9**	**1,6–2,1**	**1,9**	**1,7–2,2**	**2,0**	**1,4–2,6**	**1,9**	**1,7–2,2**
*In natura* (frescas ou secas)	1,8	1,6–2,1	1,9	1,7–2,1	2,0	1,4–2,6	1,9	1,7–2,1
Polpa^f^	0,0	0,0–0,0	0,0	0,0–0,0	0,0	0,0–0,0	0,0	0,0–0,0
**Legumes e verduras**	**0,8**	**0,7–0,9**	**0,7**	**0,7–0,8**	**0,8**	**0,7–0,9**	**0,7**	**0,7–0,8**
*In natura*	0,8	0,7–0,9	0,7	0,6–0,8	0,8	0,6–0,9	0,7	0,6–0,8
Conservas e extratos	0,0	0,0–0,0	0,0	0,0–0,0	0,0	0,0–0,0	0,0	0,0–0,0
**Café, chá, mate e especiarias**	**0,2**	**0,1–0,2**	**0,2**	**0,1–0,2**	**0,2**	**0,1–0,3**	**0,2**	**0,1–0,2**
Especiarias^g^	0,1	0,0–0,1	0,1	0,0–0,1	0,1	0,1–0,2	0,1	0,1–0,1
Café	0,1	0,0–0,1	0,1	0,0–0,1	0,1	0,0–0,1	0,1	0,0–0,1
Mate	0,0	0,0–0,0	0,0	0,0–0,0	0,0	0,0–0,0	0,0	0,0–0,0
Chá	0,0	0,0–0,0	0,0	0,0–0,0	0,0	0,0–0,0	0,0	0,0–0,0
**Castanhas e nozes** ^h^	**0,0**	**0,0–0,0**	**0,0**	**0,0–0,0**	**0,0**	**0,0–0,0**	**0,0**	**0,0–0,0**
Total	82,5	81,1–83,8	85,0	84,1–85,9	83,8	81,6–85,9	85,2	84,3–86,2

IC95%: intervalo de confiança de 95%.

*Foram avaliados somente os domicílios chefiados por pessoas pretas, pardas e brancas e as outras categorias não foram incluídas devido ao baixo tamanho amostral.

**Raça/cor da pele preta e parda agrupadas.

^a^ Feitos somente de farinha de trigo e/ou outras farinhas feitas de alimentos in natura e minimamente processados, leveduras, água, sal e/ou outros alimentos in natura e minimamente processados.

^b^ Massas frescas ou secas feitas com farinhas/sêmola, água e/ou ovos e/ou outros alimentos in natura ou minimamente processados.

^c^ Feitos de leite e sal e microrganismos usados para fermentar o leite.

^d^ Sem adição de açúcar, edulcorante e/ou aditivos que modifiquem as características sensoriais do produto.

^e^ Maniçoba, tucupi e farinha/gomo de tapioca, entre outras.

^f^ Sem adição de açúcar.

^g^ Pimenta, noz-moscada, açafrão, entre outros.

^h^ Sem sal ou açúcar.

As estimativas da participação média calórica dos alimentos da Nova Cesta Básica, detalhadas para o Brasil como um todo e para as grandes regiões, estão disponíveis em repositório público online^a^.

## DISCUSSÃO

Os alimentos que passaram a compor a cesta básica constituíram, em 2017–2018, a base da alimentação em domicílios de baixa renda em todos os estratos sociodemográficos avaliados, representando quase 85,0% das calorias consumidas. Esse valor superou 88,0% em domicílios da zona rural e da região Norte do país.

Os achados são significativos ao indicar que a nova cesta básica, além de saudável e sustentável, já reflete um padrão alimentar amplamente consumido pela população brasileira. Isso reforça a viabilidade cultural da implementação da cesta nas políticas públicas e o combate a discursos hegemônicos e equivocados, baseados no senso comum de que alimentos ultraprocessados seriam fontes importantes de alimentos em populações com maior risco de insegurança alimentar^12,13^.

Nesse sentido, destaca-se o elevado consumo do grupo de carnes e ovos nessa população, que superou 11% das calorias, similar ao da população geral (10,8% das calorias)^14^. É importante ressaltar que esse percentual se refere a proteínas de origem animal, sem considerar as de origem vegetal (como feijão), o que significa que, isoladamente, esse grupo já atinge parâmetros adequados de recomendação internacional de proteína^12^ na população de baixa renda. No entanto, é relevante pontuar que isso nem sempre é positivo, já que o consumo excessivo de carne bovina, embora seja uma fonte importante de nutrientes, está associado ao desenvolvimento de doenças como certos tipos de câncer e doenças cardiovasculares^15^, além de ter um alto impacto ambiental^18^. No Brasil, o aumento na participação da carne bovina na dieta tem sido constante nas últimas décadas, especialmente nas faixas de renda mais baixas^14^.

Por outro lado, observou-se um consumo insuficiente de frutas, verduras e legumes nessa população. Estes alimentos, cujo consumo adequado previne o ganho de peso excessivo e reduz o risco de diversas doenças crônicas não transmissíveis (DCNT), corresponderam a apenas 2,7% das calorias, menos da metade das recomendações da Organização Mundial da Saúde (pelo menos 400 gramas diárias ou cerca de 5-6% das calorias totais de uma dieta de 2.000 kcal diárias)^21^, sendo esse valor inferior à média observada para a população geral (3,7% das calorias)^14^. O consumo de açúcar, por sua vez, foi excessivo, superando 10% das calorias em todos os subgrupos avaliados, com exceção da região Nordeste. A alta ingestão de açúcares é uma preocupação de saúde pública, pois tem sido associada à piora na qualidade nutricional da dieta, ao consumo excessivo de energia e ao risco de desenvolvimento de obesidade e DCNT^22,23^.

Os resultados, que mostraram pequenas diferenças de acordo com a raça/cor da pele, corroboram com o estudo anterior que avaliou o consumo alimentar da população brasileira em 2017–2018^24^. Esse estudo descreveu que pretos e pardos apresentaram apenas ligeira maior adesão à regra de ouro do Guia Alimentar para a População Brasileira. No entanto, isso não se traduziu necessariamente em maior qualidade global da alimentação, uma vez que essa diferença se deveu quase que exclusivamente ao maior consumo de alimentos básicos como arroz e feijão, não ocorrendo o mesmo para outros alimentos *in natura* ou minimamente processados, como frutas, legumes, verduras, nozes e sementes^24^. Além disso, essas diferenças no consumo alimentar foram, em grande parte, explicadas por diferenças nas condições socioeconômicas entre estratos de raça/cor da pele^24^, sendo provável que, neste estudo, ao avaliar somente pessoas de baixa renda, essa diferença tenha se atenuado ainda mais.

No Brasil, a adesão a um padrão alimentar baseado em alimentos *in natura* ou minimamente processados é maior em populações dos estratos socioeconômicos mais baixos^25^. No entanto, estudo recente descreveu um aumento no consumo de alimentos ultraprocessados entre 2008 e 2018 na população brasileira e que ele foi mais intenso entre pessoas mais pobres, menos escolarizadas, moradores da zona rural, das regiões Norte e Nordeste, e pretos e pardos^25^. Isso indica uma padronização nacional em níveis mais elevados e com concentração de riscos nas populações mais vulnerabilizadas.

As mudanças na nova cesta básica têm, portanto, potencial para impactar positivamente na saúde da população, já que um padrão alimentar baseado em alimentos *in natura* ou minimamente processados está associado a maior qualidade nutricional da dieta e menor risco de diversas DCNT, como obesidade, diabetes e doenças cardiovasculares^26,27^. Além disso, esses alimentos, quando são de origem vegetal, têm efeitos positivos para a sustentabilidade ambiental, produzindo menor pegada de carbono e hídrica e fomentando a agrobiodiversidade^28^. Isso é ainda mais relevante ao considerarmos que as DCNT e as mudanças climáticas acometem mais intensamente as populações mais pobres e periféricas^28^.

Observaram-se diferenças regionais e entre urbano e rural nos grupos e subgrupos dos alimentos, indicando a identidade e como o consumo dos alimentos variam no Brasil. Isso corrobora com os dados de Levy et al.^14^ (2023), em que observaram diferença no consumo dos alimentos nas diferentes estratificações. Além disso, a nova cesta também estimula o consumo de alimentos regionais e da sociobiodiversidade, importantes para a cultura, economia local e meio ambiente^5,29^. Isso é crucial, considerando que os alimentos regionais representaram apenas 3,12% da participação calórica em 2017–2018, com uma queda significativa desde 2002 nos tubérculos, raízes e cereais regionais^30^. Em relação aos alimentos da sociobiodiversidade, com exceção da erva-mate, a disponibilidade é baixa, representando uma média de 7,09g/*per capita*/dia em 2017–2018^31^.

A cesta básica tem grandes implicações ao servir como um marco normativo para a produção de políticas que favoreçam a produção e o acesso a alimentos saudáveis e sustentáveis no Brasil. No novo plano Safra 2024–2025, por exemplo, houve uma redução nas taxas de juros para agricultores que produzem alimentos da nova cesta básica, além de produtos da sociobiodiversidade, agroecológicos e orgânicos^32^. O novo decreto da cesta básica também desempenha um papel central na discussão da reforma tributária que está em curso, na qual o governo propõe que os seus alimentos tenham alíquotas zero ou reduzidas. Isso é ainda mais importante ao considerarmos que a população de baixa renda gasta quase metade de seus rendimentos na aquisição de alimentos^33^ e que o preço relativo de alimentos ultraprocessados vem reduzindo substancialmente desde o início dos anos 2000, tornando-se mais baratos que alimentos *in natura* ou minimamente processados^34^.

Este estudo possui algumas limitações, incluindo as análises de raça/cor da pele, em que não foram incluídos os domicílios chefiados por pessoas que se autodeclaram amarelas e indígenas devido à baixa representatividade dessas populações na POF, principalmente quando a análise é estratificada apenas para os domicílios de baixa renda. Outra limitação é o fato de o inquérito de aquisição não considerar desperdício, consumo de alimentos fora de casa e divisão intrafamiliar do consumo, o que pode levar a vieses na estimativa do que de fato foi consumido nos domicílios. Além disso, o inquérito data de 2017–2018 e o cenário pode ter se alterado, especialmente no período pós-pandemia. No entanto, a POF 2017–2018 é o inquérito nacional mais recente com representatividade nacional e que possui alto nível de detalhamento, o suficiente para identificar os alimentos estudados, além de representar a maior parte do consumo, já que a alimentação fora de casa representa uma parte menor^35^. Ademais, este estudo pode servir como linha de base para identificar tendências temporais e avaliar os potenciais impactos gerados por esse decreto de 2024 a partir de novos inquéritos domiciliares. Destaca-se que este estudo é inovador ao focar na população usualmente invisibilizada nos estudos e por incluir a variável de raça/cor da pele, geralmente subutilizada.

Em conclusão, nossos dados indicam que, em 2017–2018, os alimentos que passaram a compor a nova cesta básica já eram a base da alimentação dos domicílios em situação de baixa renda.

Contudo, também foi observado inadequações na dieta dessa população, como o alto consumo de carne bovina, o baixo consumo de frutas, legumes e verduras e o consumo excessivo de açúcar. Assim, reforça-se a viabilidade cultural de implementar uma dieta saudável e sustentável alinhada à proposta da cesta, mas ressalta-se que o impacto dessa implementação seria significativamente ampliado se, além do acesso aos alimentos, houvesse ações combinadas que garantissem a priorização de alimentos *in natura* ou minimamente processados de origem vegetal e que apoiassem a população a fazer escolhas mais saudáveis.

## Data Availability

Este estudo utilizou dados secundários de acesso público, que não contêm informações capazes de identificar os participantes. https://www.ibge.gov.br/estatisticas/sociais/populacao/24786-pesquisa-de-orcamentosfamiliares-2.html?=&t=microdados

## Footnotes

a Disponível em: https://doi.org/10.17605/OSF.IO/MU2BR
